# Hydrodechlorination of 4-Chlorophenol on Pd-Fe Catalysts on Mesoporous ZrO_2_SiO_2_ Support

**DOI:** 10.3390/molecules26010141

**Published:** 2020-12-30

**Authors:** Ekaterina S. Lokteva, Vera V. Shishova, Nikolay N. Tolkachev, Andrey N. Kharlanov, Konstantin I. Maslakov, Alexey O. Kamaev, Igor Yu. Kaplin, Irina N. Savina, Elena V. Golubina

**Affiliations:** 1 Chemistry Department, Lomonosov Moscow State University, Leninskie Gory 1/3, 119991 Moscow, Russia; vshishova@bk.ru (V.V.S.); kharl@kge.msu.ru (A.N.K.); Nonvitas@gmail.com (K.I.M.); kamaevalexey@gmail.com (A.O.K.); kaplinigormsu@gmail.com (I.Y.K.); golubina@mail.ru (E.V.G.); 2 N.D. Zelinsky Institute of Organic Chemistry, Russian Academy of Sciences, Leninsky Prospect 47, 119991 Moscow, Russia; nick33@bk.ru; 3 School of Pharmacy and Biomolecular Sciences, University of Brighton, Huxley Building, Lewes Road, Brighton BN2 4GJ, UK; i.n.savina@brighton.ac.uk

**Keywords:** hydrodechlorination, 4-chlorophenol, phenol, bimetallic catalyst, iron, palladium, zirconium oxide, silica, mild reduction

## Abstract

A mesoporous support based on silica and zirconia (ZS) was used to prepare monometallic 1 wt% Pd/ZS, 10 wt% Fe/ZS, and bimetallic FePd/ZS catalysts. The catalysts were characterized by TPR-H_2_, XRD, SEM-EDS, TEM, AAS, and DRIFT spectroscopy of adsorbed CO after H_2_ reduction in situ and tested in hydrodechlorination of environmental pollutant 4-chlorophelol in aqueous solution at 30 °C. The bimetallic catalyst demonstrated an excellent activity, selectivity to phenol and stability in 10 consecutive runs. FePd/ZS has exceptional reducibility due to the high dispersion of palladium and strong interaction between FeOx and palladium, confirmed by TPR-H_2_, DRIFT spectroscopy, XRD, and TEM. Its reduction occurs during short-time treatment with hydrogen in an aqueous solution at RT. The Pd/ZS was more resistant to reduction but can be activated by aqueous phenol solution and H_2_. The study by DRIFT spectroscopy of CO adsorbed on Pd/ZS reduced in harsh (H_2_, 330 °C), medium (H_2_, 200 °C) and mild conditions (H_2_ + aqueous solution of phenol) helped to identify the reasons of the reducing action of phenol solution. It was found that phenol provided fast transformation of Pd^+^ to Pd^0^. Pd/ZS also can serve as an active and stable catalyst for 4-PhCl transformation to phenol after proper reduction.

## 1. Introduction

Chlorinated organic substances have xenobiotic nature, many of them are toxic, carcinogenic, bioaccumulative, and mimic the structure of mammalian hormones [1]. Despite the ban on the production of many highly toxic chlorinated persistent organic pollutants (POPs) under the Stockholm Convention (2001), organochlorine compounds continue to be used in industry as solvents, starting reagents for the manufacture of polymers, in the composition of fire-resistant materials, medicines, herbicides, insecticides, etc. Over the past few years, a significant amount of these substances has been accumulated in nature in the composition of soils, sedimentary rocks, water bodies, and groundwater [2], and due to climate change they are redistributed across the planet and reach remote locations where direct industrial pollution does not occur [3]. Therefore, in recent years, the focus of environmental concerns has shifted towards the decontamination of aqueous solutions from chlorinated POPs.

Chlorophenols are the class of chlorinated POPs that are still widely used in many industries—as intermediates in organic synthesis, in plant protection products, as components of dyes, disinfectants, wood preservatives, etc. [4]. At the same time, they are toxic and hardly degradable products [5]. Therefore, the development of the useful methods for their decomposition is of an urgent need.

Hydrodechlorination (HDC) was proven to be environmentally benign method of chlorinated POPs disposal because it excludes the formation of dangerous oxidation products such as dioxins and phosgene, provides transformation of chlorinated organics to the commercially valuable products [1,6,7,8], and can be performed in relatively mild conditions. It was successfully used in the water phase to process chlorinated phenols [9,10,11,12,13] and medicine components [14,15]. The catalytic HDC of chlorophenols is an attractive way of their transformation, because it can be realized both for concentrated and diluted solutions, in mild conditions using benign reductant—hydrogen, to produce phenol, which is safer than chlorinated phenols, and it is in demand in several chemical processes, such as the production of pesticides, dyes, resins, lubricants, solvents, etc. [16].

Palladium is the most attractive active component of the HDC catalysts due to its high efficiency and stability [17,18,19]. However, bimetallic Pd-containing catalysts often outperform monometallic palladium catalysts in their efficiency mainly due to the formation of mixed active sites, enhanced dispersion of the active phase, while the decrease in the content of the noble metal reduces the cost of the catalyst. Iron is a very promising modifier for Pd because of its low cost, abundance in the Earth, and high efficiency of iron-containing bimetallic catalyst [13,20,21]. Our early work demonstrated the improved catalytic properties of FePd/C catalysts in the multiphase HDC of 1,4-dichlorobenzene, hexachlorobenzene, and 2,4,8-trichlorodibenzofurane [20] compared to monometallic Pd/C. The strong interaction between metals, demonstrated by temperature-programmed reduction with H_2_ (TPR-H_2_) and X-ray photoelectron spectroscopy (XPS), the formation of Pd-enriched PdFe alloy particles and prevention of Pd chlorination under protective action of Fe were proposed to be the main reasons for the enhanced activity of the bimetallic catalyst.

The significant interest in the HDC of chlorophenols is evident from the large number of publications on this topic. Thus, Pd-containing mono- and bimetallic catalysts were used for the HDC of chlorophenols [5,7,8,10,11,21,22,23,24,25,26,27,28,29,30].

The nature of support for Pd-containing catalysts plays an important role in providing high catalyst efficiency in the HDC. Thus, SiO_2_ and Al_2_O_3_ [21], resins [30], pillared clays [31], carbon nanosheets [32], reduced graphene oxide [33], mesoporous silica–carbon nano-composite [22], and many other supports were investigated. The ordered mesoporous structure of a support can be an advantage for catalysts in various reactions providing high specific surface area, tunable pore sizes, facilitating the diffusion of reactants and products inside pores [34]. Zirconium dioxide is known as a chemically and thermally stable support for metal-containing catalysts. Although the possibility of the synthesis of ordered mesoporous zirconium dioxides with high specific surface value was demonstrated [35], the simple and large-scale production of the zirconia with the specific surface area higher than 200 m^2^/g remains a challenge. Modification of zirconia with silica is a promising way to overcome this shortcoming and produce the highly ordered mesoporous structure with the high specific surface area [36,37]. Such supports were successfully used for the catalysts of syngas transformation to alcohols [38], dry reforming of methane to syngas [39], etc. However, mixed silica–zirconia supports have never been tested as supports for the HDC catalysts.

This work focuses on the synthesis of the monometallic Pd and Fe and bimetallic PdFe catalysts on the novel mesoporous ZrO_2_SiO_2_ support. Their performance in the hydrodechlorination of 4-chlorophenol in water solution was compared. Three catalysts (Pd/ZrO_2_SiO_2_, Fe/ZrO_2_SiO_2_, FePd/ZrO_2_SiO_2_) were synthesized with the 1 and 10 wt% of Pd and Fe contents, respectively. It was expected that the mesoporous structure and high specific surface of the support together with the strong interaction between iron and palladium in the bimetallic catalyst will enhance the catalyst properties.

## 2. Results

### 2.1. Texture and Morphology

The porosity and morphology of the catalysts were studied by N_2_ physisorption and scanning electron microscopy (SEM) combined with energy-dispersive X-ray spectroscopy (EDS).

**Figure 1:**
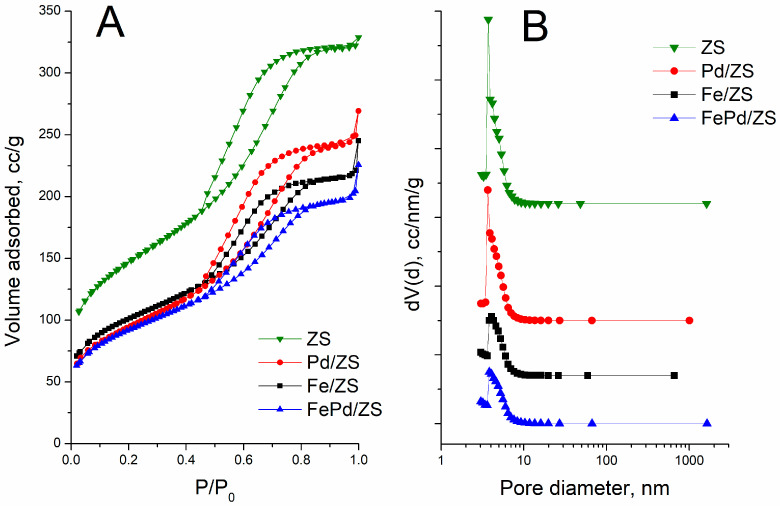
Nitrogen physisorption isotherms (**A**) and pore size distributions (**B**) for unreduced catalysts.

The low temperature N_2_ physisorption isotherms of the support and unreduced catalyst (Figure 1) can be attributed to Type IV(a) according to the IUPAC classification [40] with the well-pronounced hysteresis caused by capillary condensation in mesopores.

The support has the remarkably high S_BET_, 529 ± 53 m^2^/g (Table 1), confirming the formation of mesoporous structure with up to 30% micropore contribution. This is not typical for ZrO_2_ prepared by commonly used methods, and resulted from the modification of zirconia with silica, which is known to form ordered mesoporous materials in the presence of the CTAB template [41,42]. The support has a monomodal pore size distribution with the average mesopore diameter of 3.7 nm (Figure 1B).

The S_BET_ values were nearly similar for all unreduced catalysts (Table 1) and they were significantly lower than that of the original support. At the same time, the shapes of hysteresis loops (Figure 1A) and pore size distributions (Figure 1B) are similar for the support and catalysts. Therefore, the decrease in S_BET_ values was primarily from the complete filling or blocking of the part of the pores rather than from the decrease in the pore size. The height of the hysteresis loop was less affected by metal deposition in the case of Pd/ZS. This could be a result of the low content of the metal (Pd) in this catalyst, that limits its ability to fill or completely block mesopores.

The partial destruction of the porous structure because of the action of the palladium nitrate solution with an extremely low pH can be another reason for the S_BET_ decrease after deposition of palladium. The iron nitrate solution, due to its lower acidity, affects the support during impregnation to a lesser degree. However, the iron oxide formed during calcination of the precursor introduces its own pores and, due to the significant iron content (10 wt%), they contribute significantly to the textural properties of the iron-containing catalysts. Thus, the iron-containing catalysts show slightly larger pore size and noticeably smaller pore volume compared to ZS, while they are nearly the same in Pd/ZS and ZS. The combined action of these factors leads to the approximately the same S_BET_ values for all three catalysts.

The metal contents measured by atomic-absorption spectroscopy (AAS, Table 2) were slightly lower than the expected values. However, the Fe/Pd wt. ratio of 9.2 in the bimetallic catalyst was close to the target value of 10.

**Figure 2:**
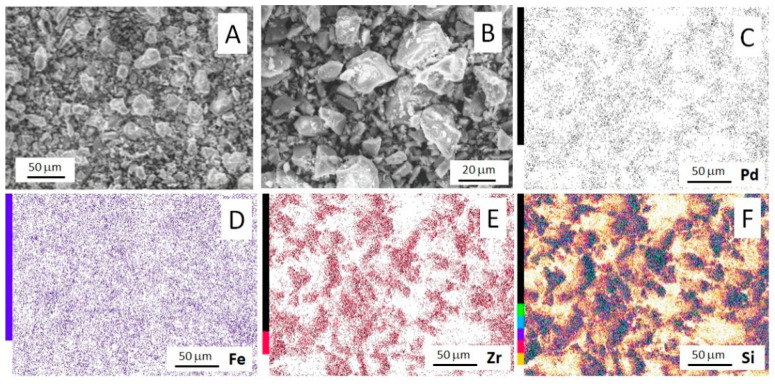
SEM images (**A**,**B**) and EDX elemental maps (**C**–**F**) for PdFe/ZS after reduction with H_2_ at 320 °C.

The morphology of the catalysts was studied by SEM-EDS (Figure 2 and Figure S1 in Supplementary Material).

The SEM images of all unreduced catalyst were similar. They demonstrated particles of irregular shapes and different sizes, but less than 50 µm. All metals (Zr, Si, Pd, and Fe) were predominantly uniformly distributed on the surface of all the samples (Figure 2 and Figure S1 in Supplementary Material).

The presence of O, Si, Zr, and C was detected by EDS in all samples (Table 2). Carbon can result from both the CO_2_ adsorption on the surface and from the adhesive tape used to fix powder samples. That is why its content (about 22–27 at %) was not presented in Table 2. The uniform distribution of Si and Zr (Figure 2, Figure S1 in Supplementary material) reflects the uniform distribution of SiO_2_ and ZrO_2_ in the support. The surface of catalysts was obviously enriched with silica. The Si:Zr atomic ratio was more than 16 in all the catalysts (Table 2), which was significantly higher than the target ratio of 1.2. At the same time, the content of the both active metals on the surface was slightly lower than expected values (0.7 wt% of Pd in Pd/ZS, 0.6 wt% of Pd in FePd/ZS, 6.1–6.2 wt% of Fe in FePd/ZS and Fe/ZS), but their weight ratio was close to the target value (Pd:Fe = 1:10). It can be noted that the iron concentration on the surface is higher than that of zirconium.

### 2.2. X-ray Diffraction (XRD) and Transmision Electron Microscopy (TEM) Analysis

**Figure 3:**
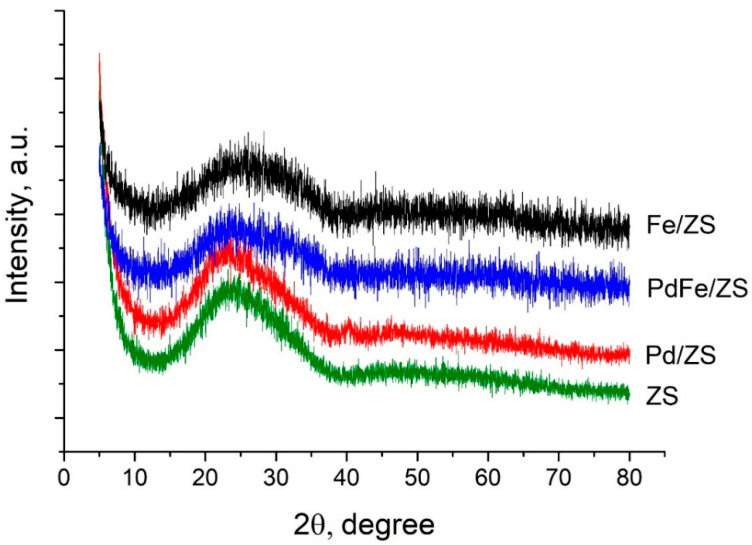
XRD profiles of ZS support and catalysts reduced in H_2_ flow for 2 h at 320 °C (Pd/ZS and FePd/ZS) or 520 °C (Fe/ZS).

Both the ZS support and the reduced catalysts were analyzed by XRD (Figure 3). The signal-to-noise ratio was very low in all diffraction patterns, which indicates the low degree of crystallinity of the samples.

An asymmetric amorphous halo at approximately 24–26° was observed in all diffraction patterns. It originated mostly from the amorphous silicon dioxide, but the contribution from amorphous ZrO_2_ was also possible. The diffraction pattern of Pd/ZS additionally showed a broad low intensive peak at approximately 40° that may be attributed to metallic Pd (JCPDS-ICDD card no. 46-1043). The absence of this reflection in the FePd/ZS pattern indicates a smaller size of Pd particles in this sample or the formation of amorphous phase comprising Pd and Fe. The iron-containing phases were not detected in XRD patterns because of their amorphous nature or extremely small crystallite size.

TEM images of the catalyst after the mild reduction with H_2_ at RT (FePd/ZS) or after the co-action of H_2_ and water solution of phenol (monometallic catalysts) were obtained (Figures S2–S4, Supplementary Material). Such treatment led to a high activity of the palladium-containing catalysts in the HDC of 4-PhCl (see below). Only a few crystalline palladium particles of 3–5 nm in size were observed in the TEM images of both Pd/ZS (Figure S2) and FePd/ZS (Figure S3), this was not enough to calculate the reliable particle size distributions. However, the number of the detected Pd particles in Pd/ZS was slightly higher than in FePd/ZS. This indirectly confirms a higher dispersion of the palladium in the bimetallic catalysts observed by XRD. Large (more than 20 nm in diameter) crystalline domains of iron oxide were observed in the several TEM images of Fe/ZS (Figure S4), but such domains were not detected in other images.

### 2.3. Reducibility of Metals in Catalysts

**Figure 4:**
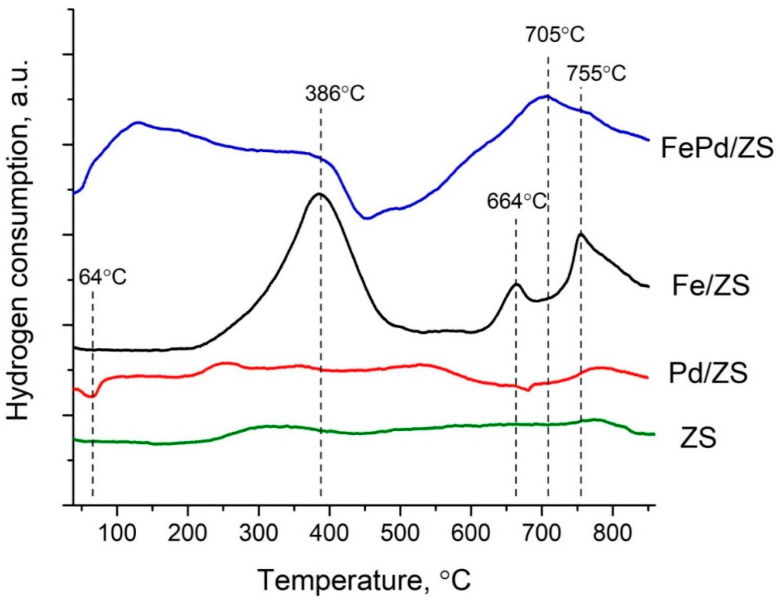
TPR-H_2_ profiles of the ZS support and unreduced catalysts.

The reducibility of the metals and the extent of metals interaction in the bimetallic catalysts were studied by TPR-H_2_. The TPR-H_2_ profiles of the unreduced catalyst and the support are presented in Figure 4.

The TPR-H_2_ profile of the support showed nearly no peaks, because both ZrO_2_ and SiO_2_ are reduced at high temperatures (more than 900 °C) [39]. Only slight H_2_ uptakes were observed at 250–400 °C and at 770–800 °C that can be attributed to the reduction of surface functional groups or to dihydroxylation [43,44].

A negative H_2_ uptake was observed in the TPR-H_2_ profile of Pd/ZS at 64 °C. This peak can be attributed to the decomposition of β-PdH_x_ that is easily formed under dissolution of H_2_ in the metallic palladium. The molar ratio of Pd loading to the released hydrogen, Pd/H_2_, was 7:1. The presence of this peak demonstrates: (i) the possibility of at least partial PdO reduction at low temperatures (less than 64 °C) under the preliminary treatment of the sample in the TPR-H_2_ instrument, and (ii) the presence of relatively large Pd particles (more than 5 nm in size), because smaller particles cannot absorb hydrogen in the bulk [45,46]. The solubility of H_2_ in palladium particles becomes negligible when their dispersity approaches to 100% [46]. The TPR-H_2_ data agrees with the small 5 nm size of Pd particles in Pd/ZS sample detected by XRD and TEM.

The TPR-H_2_ profile of Pd/ZS also have broad low intensive peaks at 250–350 °C and 550 °C. These peaks indicate the presence of different types of PdO particles with the different degree of interaction with the support. The peak at 558 °C can be assigned to the reduction of PdO_x_ species strongly interacting with the support [47], or blocked in the closed pores. Therefore, at least part of Pd can be reduced at RT, while the temperature of about 300 °C was sufficient to reduce the major portion of PdO species in this sample.

The reduction of α-Fe_2_O_3_ is known to proceed into three steps [48]. For this reason, the TPR-H_2_ profile of Fe/ZS showed an intensive peak at 386 °C with a noticeable shoulder at the low temperature side, and two high-temperature peaks at 664 and 755 °C. The reduction of Fe^3+^ proceeded accordingly to the following scheme.

Fe_2_O_3_ → Fe_3_O_4_ → FeO → Fe

The first broad peak in the TPR profile of Fe/ZS with the maximum at 386 °C corresponded to the stepwise reduction of Fe^3+^ to Fe^2+^ [49]:

Step 1: 3Fe_2_O_3_ + H_2_ → 2Fe_3_O_4_ + H_2_O
Step 2: Fe_3_O_4_ + H_2_ → 3FeO + H_2_O

The overlapping of the temperature intervals of consecutive iron reduction steps is highly probable especially when the heating rate is high [49]. Thus, the low temperature shoulder of the peak at 386 °C could be attributed to the first iron reduction step. The lower intensity of this peak compared to the second overlapped peak agrees with the stoichiometry of the reduction steps.

The reduction of Fe^2+^ to Fe^0^ can proceed only at high temperatures. Therefore, the peaks at 664 and 755 °C can be attributed to the reduction of FeO on the surface and in the bulk of the material, in smaller or larger particles, weakly or strongly interacting with the support [50].

Step 3: FeO + H_2_ → Fe + H_2_O

The TPR-H_2_ data demonstrated that iron in the unreduced Fe/ZS existed as Fe^3+^ species and they can be relatively easily reduced with H_2_ to Fe^2+^ at approximately 400 °C, but further transformation to Fe^0^ can be possible only at temperatures higher than 664 °C. The results agreed with the earlier in situ XRD study, in which a non-supported hematite powder (α-Fe_2_O_3_) was reduced to a magnetite (Fe_3_O_4_) in extremely diluted H_2_/He mixture at 400 °C [51]. At the higher temperature of about 600 °C a mixture of wüstite (FeO) and Fe^0^ was observed in this study, and only Fe^0^ reflexes were detected at 700 °C. The reduction of supported iron species should certainly proceed at slightly higher temperatures.

The TPR-H_2_ profile of the bimetallic catalyst was quite different from those of the monometallic ones. It had a broad band of poorly resolved overlapped peaks from approx. 50 to approx. 450 °C with two vague maximums at about 130 and 386 °C. Another broad peak with the maximum at about 705 °C was observed in the high temperature range from 450 to 900 °C. Such a broad TPR-H_2_ profile indicated the strong interaction of metals in the bimetallic catalyst, which shifts the Fe^3+^ reduction to the lower temperatures. The high-temperature peaks of PdO reduction were shifted to the lower temperature indicating easier reduction of the palladium particles. The position and shape of the high-temperature peak confirmed that Pd^0^ species shifted FeO to Fe^0^ reduction to the lower temperatures. Earlier it was reported that the modification of iron oxides with palladium and zirconium oxides led to a synergetic effect in the decrease of the reduction temperature of iron oxides to metal [52]. The suppression of sintering of palladium particles was proposed to be a reason for the promoting action of zirconia or zirconia-containing supports. Such supports provided high stability of Pd/ZrO_2_ catalysts in methane combustion [53,54].

In contrast to Pd/ZS, no negative H_2_ uptake of PdH_x_ decomposition was observed in the TPR-H_2_ profile of FePd/ZS probably because of the higher Pd dispersion in the latter sample. This fact confirmed the assumption about higher Pd dispersion in the bimetallic catalyst, made on the base of the XRD and TEM data.

The TPR-H_2_ data for the unreduced catalyst demonstrated that the reduction of the catalysts with molecular H_2_ requires high temperatures, which can lead to the sintering of the metal particles and changes in the porous structure of the support with the simultaneous decrease of the pore volume and pore sizes. Therefore, the preliminary reduction using other methods, such as NaBH_4_ treatment, seems to be more attractive.

### 2.4. Diffuse Reflectance Infrared Fourier Transform Spectroscopy (DRIFT) Study of Adsorbed CO

**Figure 5:**
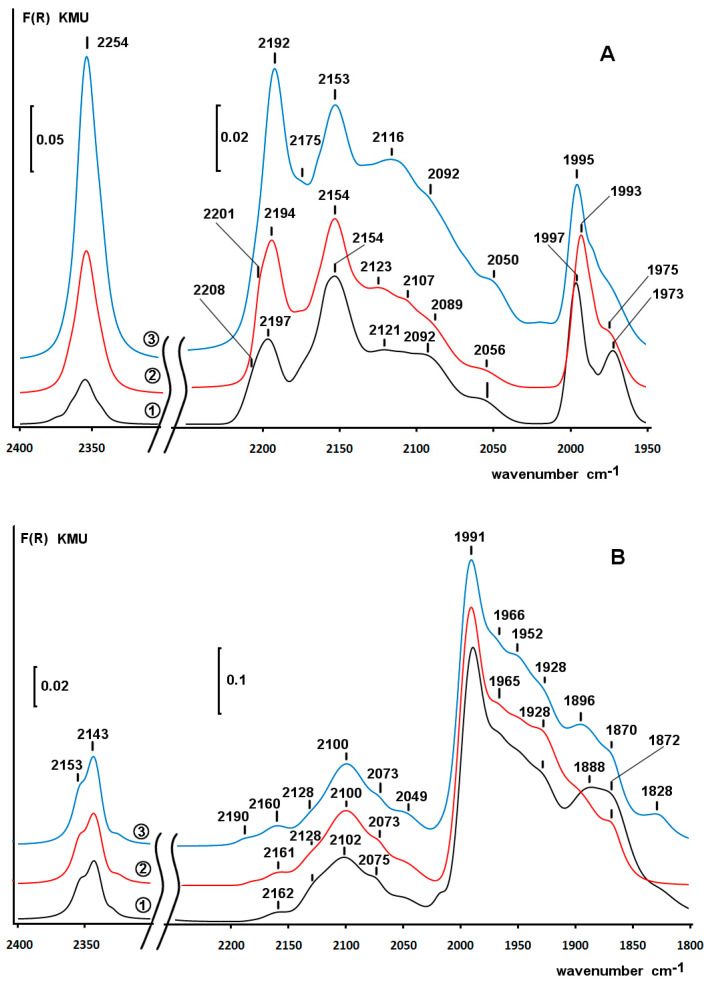
Difference DRIFT spectra of CO adsorbed at room temperature on Pd/ZS(550) before (**A**) and after H_2_ treatment at 330 °C (**B**). CO pressure: 5 Torr—curve 1, 20 Torr—curve 2, 50 Torr—curve 3. F(R)—Kubelka–Munk function (Kubelka–Munk units).

The catalysts were studied by DRIFT spectroscopy of adsorbed CO before and after reduction with H_2_. Figure 5 presents the data for Pd/ZS. Firstly, the unreduced catalysts calcined at 550 °C (denoted as Me/ZS(550)) were analyzed. Such calcination is a common way to remove surface functional groups.

A superposition of several absorption bands was observed in the spectrum of CO, adsorbed on Pd/ZS(550) (Figure 5A). The bands at 2192–2197 cm^−1^ with the shoulder at 2201–2208 cm^−1^, and a weak band at 2175 cm^−1^ were attributed to (Pd^2+^)–CO complexes. The bands at 2153–2154 cm^−1^ and the broad asymmetric band at 2116 cm^−1^ can be assigned to linear CO complexes of oxidized palladium (Pd^+^–CO) [55].

The band at 2089–2092 cm^−1^ corresponded to linear Pd^0^–CO complexes, and the band at 2050–2056 cm^−1^ to linear CO complexes of palladium atoms localized on the edges, steps, and terraces of (100) and (111) faces [56,57]. Large full widths at half maximum (FWHM) of these absorption bands and the band at 2116 cm^−1^ indicated the non-uniform palladium surface. A weak broad band at 1980 cm^−1^ can be assigned to carbonyl (Pd^0^)_2_–CO complexes [56,57,58].

Summarizing, the surface of this sample contained predominantly Pd^2+^ and Pd^+^ cations, while the contribution from Pd^0^ was low. In the high-frequency region of the spectrum the absorption band at 2354 cm^−1^ was observed. This band appeared for CO_2_ molecules coordinated on the surface without the formation of carbonate. The adsorption sites for these molecules were questionable in the literature but it can be assumed that they may include palladium cations. The observed band can be attributed to CO_2_ complexes of metallic Pd (Pd^0^… CO_2_) [59]. The appearance of CO_2_ complexes pointed to the presence of the surface reactive sites capable of oxidizing CO at room temperature.

The in situ treatment of the Pd/ZS(550) with H_2_ at 330 °C led to the significant changes in the DRIFT spectra of the adsorbed CO (Figure 5B), as result of the reduction of the most part of the palladium species to the metallic Pd. The absorption bands of linear Pd^0^–CO complexes at 2100–2102 and 2073–2075 cm^−1^ and the absorption bands of bridged adsorption of CO on Pd^0^ atoms at 1991, 1965, 1928–1934, and 1870–1872 cm^−1^ were mainly observed on the spectrum. There was only a small contribution from the bands of oxidized Pd: at 2160–2162 cm^−1^ of Pd^+^–CO complexes and at 2190–2194 cm^−1^ of Pd^2+^–CO complexes. These data confirmed the complete PdO reduction with H_2_ at about 330 °C observed by the TPR-H_2_.

**Figure 6:**
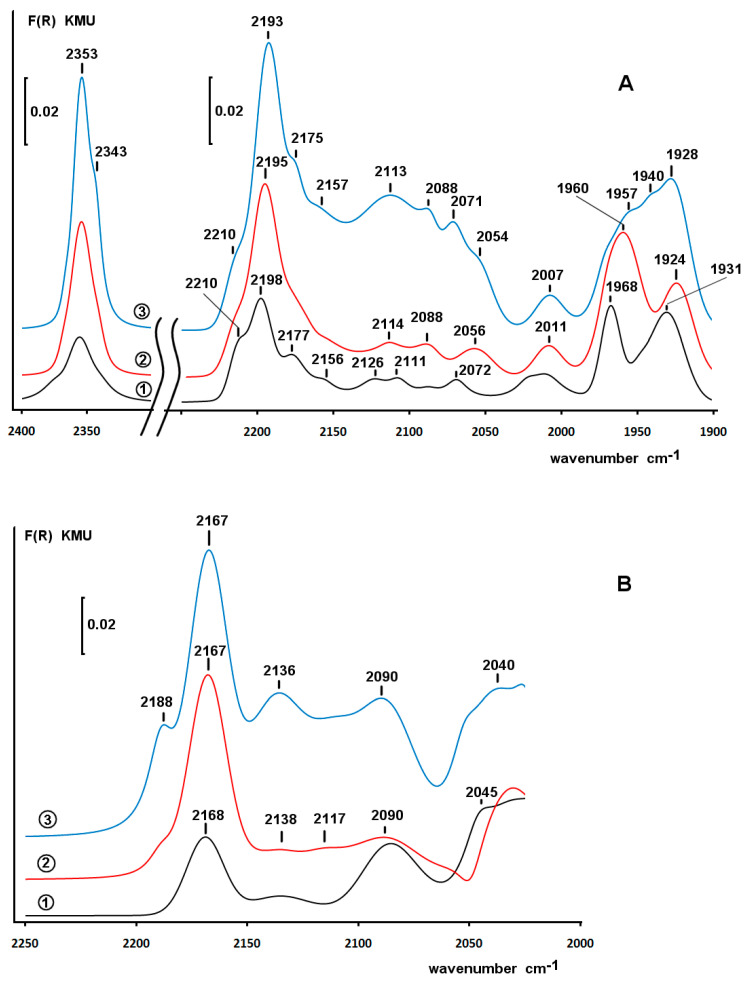
Difference DRIFT spectra of CO adsorbed at room temperature on Fe/ZS(550) before (**A**) and after H_2_ treatment (**B**). CO pressure: 5 Torr—curve 1; 20 Torr—curve 2; 50 Torr—curve 3. F(R)—Kubelka–Munk function (Kubelka–Munk units).

Fe^3+^ ions were coordinately saturated and did not form complexes with weak electron donors such as CO molecule [59,60,61]. Therefore, all peaks in the high-frequency range of the spectra of CO adsorbed on Fe/ZS(550) (Figure 6A) can be attributed to the carbonyl complexes of Fe^2+^.

According to the data from [62] refined by quantum chemical calculations using the DFT method [63], the bands at 2192–2194 and 2175–2177 cm^−1^ referred to the linear adsorption of CO on [FeOFe]^2+^ and [FeO]^2+^ centers, respectively. The band at 2175 cm^−1^ was attributed to the dicarbonyl [59] or tricarbonyl [64] forms of the CO adsorption. The absorption band at 2210 cm^−1^ noticeable only at a low surface coverage with CO molecules was related to CO complexes of isolated Fe^2+^ cations.

Several poorly resolved bands were overlapped at about 2113 cm^−1^. In the spectra recorded at low surface coverages with CO molecules, the absorption bands were observed at 2111–2114, 2085–2088, and 2071–2072 cm^−1^ and they can be attributed to carbonyl complexes of Fe^2+^ cations on the surface of the metallic iron particles. These complexes were different in the nearest environment of Fe^2+^ ions. The 2085 cm^−1^ band corresponded to CO coordination with Fe^2+^ cations surrounded by Fe^0^ atoms on the surface of the metallic iron particle. The band at 2110 cm^−1^ was attributed to Fe^2+^ cations coordinated with oxygen atoms and the band at 2071–2072 cm^−1^ was assigned to Fe^2+^ cations coordinated only with Fe^0^ atoms [65]. These adsorption sites appeared via oxidation of surface Fe^0^ species. The low-frequency band at 2054–2056 cm^−1^ corresponded to linear adsorption of CO on Fe^0^ atoms.

The band at 2353–2355 cm^−1^ in the high-frequency region of the spectrum corresponded to the coordination of CO_2_ molecules on the surface without carbonate formation, most probably on Fe^0^ atoms.

The DRIFT data demonstrated the presence of both Fe^0^ and oxidized iron species in the catalysts after a thermal-vacuum treatment, but metallic iron was partially covered with oxidized species.

After reduction in H_2_, the optical characteristics of the Fe/ZS(550) deteriorated significantly (Figure 6B), which indicated the reduction of most part of iron. The spectrum of adsorbed CO was dominated by the band at 2167 cm^−1^ with a shoulder at 2188 cm^−1^ that can be attributed to CO adsorption on [FeOFe]^2+^ and [FeO]^2+^ centers, respectively. The shift of the band position relative to the oxidized sample was caused by the influence of the reduced iron phase or by its strong interaction with the support. The band with a maximum at 2090 cm^−1^ was a superposition of several bands of CO complexes of Fe^2+^ cations on the surface of Fe^0^ particles. The band at 2040–2045 cm^−1^ and the bands in the lower frequency range can be attributed to CO complexes of Fe^0^ and sub-carbonyl forms of CO adsorption. Therefore, after reduction under severe conditions (330 °C) iron partly remained on the surface as Fe^2+^ cations, but in a different species than in the oxidized sample. Higher temperatures were required to achieve a complete iron reduction.

**Figure 7:**
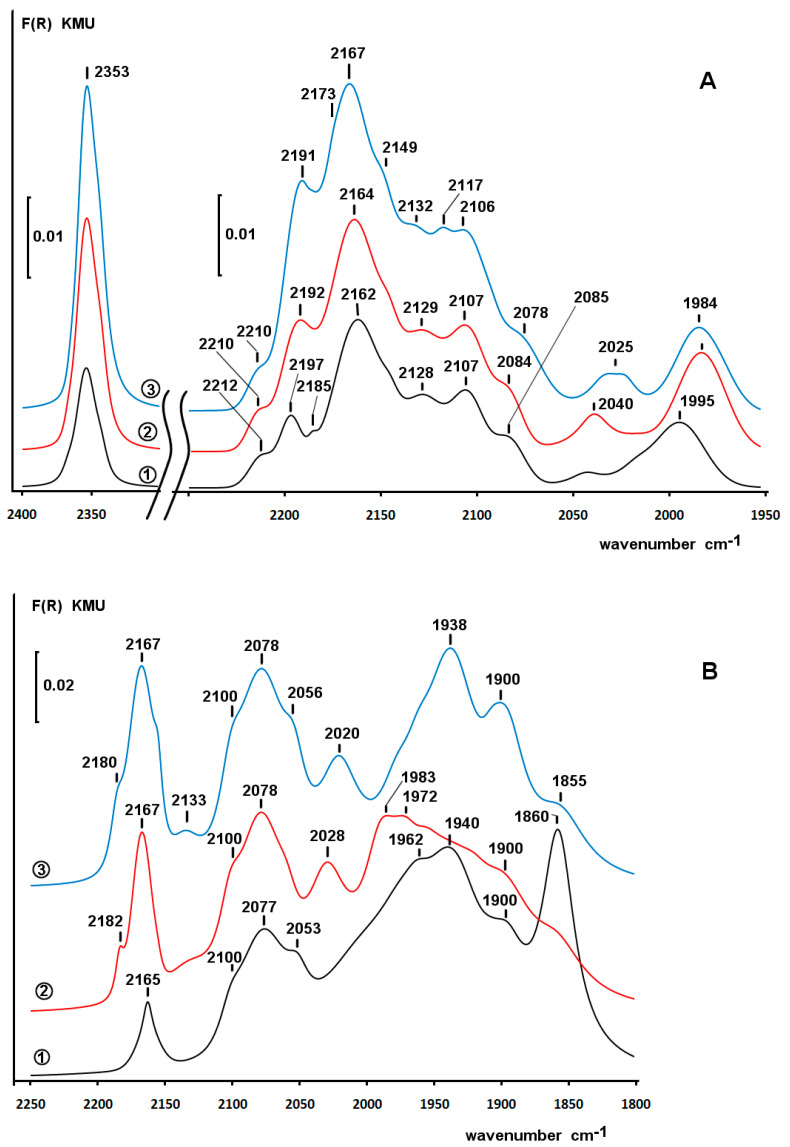
Difference DRIFT spectra of CO adsorbed at room temperature on the FePd/ZS(550) before (**A**) and after H_2_ treatment (**B**). CO pressure: 5 Torr—curve 1; 20 Torr—curve 2; 50 Torr—curve 3. F(R)—Kubelka–Munk function (Kubelka–Munk Units).

The spectrum of CO adsorbed on FePd/ZS(550) (Figure 7A) showed a superposition of absorption bands for Pd and Fe. The bands at 2191–2197 and 2185 cm^−1^ can be assigned to CO complexes of both Pd^2+^ and Fe^2+^; but the attribution of these bands to a particular metal was complicated in this case. The poorly resolved band at 2173 cm^−1^ overlapped with the more intense band at 2162–2167 cm^−1^ was attributed to the linear CO adsorption on [FeO]^2+^ centers. The superposition of several poorly resolved bands at 2128–2132, 2117, 2106–2107, and 2078–2085 cm^−1^ was related to the formation of CO complexes with palladium and iron atoms. In the frequency range below 2000 cm^−1^, the bands between 1984 and 1995 cm^−1^ were observed and attributed to sub-carbonyl complexes of Fe^0^. The low-frequency band at 1980 cm^−1^ was attributed to sub-carbonyl complexes of Fe^0^.

Interestingly, the spectrum is dominated by the broad band at 2162–2167 cm^−1^ that can be assigned to linear complexes of CO with oxidized palladium (Pd^+^–CO) [55]; but a similar band was observed in the spectra of Fe/ZS(550) after reduction with H_2_ (Figure 6B). Probably this band in the spectrum of FePd/ZS(550) is a superposition of several overlapping adsorption bands. Therefore, it can be assumed that both types of complexes existed on the surface. Note that Pd and Fe species were partially reduced in these complexes. Therefore, FePd/ZS(550) was much better reduced under the conditions of thermal vacuum treatment than both monometallic samples. This indirectly confirmed the interaction of Pd and Fe in the catalyst. Summarizing, FePd/ZS(550) comprised both oxidized and reduced forms of Pd and Fe.

After H_2_ treatment of the FePd/ZS(550), the optical characteristics deteriorated significantly due to the formation of large amount of Fe^0^ (Figure 7B). The spectrum of adsorbed CO showed a band at 2078 cm^−1^ that corresponds to linear adsorption of CO on Fe^0^, and a broad superposition of poorly resolved band with the maximum at 1938 cm^−1^ related to adsorbed sub-carbonyl Fe^0^_n_(CO)_m_ species [59]. The shoulder at 2100 cm^−1^ of the 2078 cm^−1^ band corresponds to complexes of CO with Pd^0^.

The band at 2167 cm^−1^ was well resolved and narrower than that in the spectrum of the oxidized sample. We believe that this band in the spectrum of the reduced catalysts corresponds mainly to the CO adsorption on [FeO]^2+^ centers.

The DRIFT results indicated a high reducibility of the metals in FePd/ZS(550). It seems that palladium was totally reduced to Pd^0^. The strong difference in the surface forms of CO adsorption on FePd/ZS(550) compared to both Pd/ZS(550) and Fe/ZS(550) was an evidence of the noticeable changes in the surface properties as a result of the interaction between palladium and iron. The DRIFT data mainly agreed with the TPR-H_2_ results.

### 2.5. Catalytic Tests in 4-PhCl Reduction

The ZS support demonstrated no activity in the 4-PhCl transformation at the reaction conditions.

**Figure 8:**
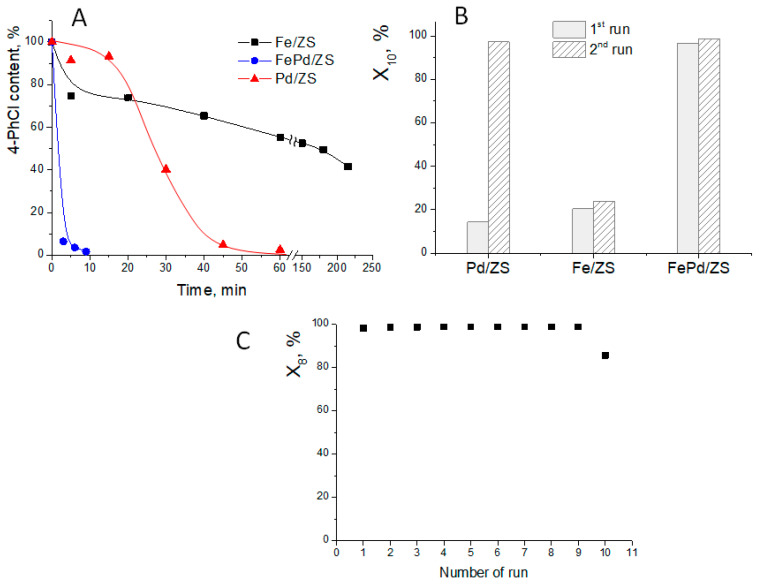
4-PhCl content in the reaction mixture vs. reaction time over the catalyst reduced with NaBH_4_ +H_2_ (**A**); conversion of 4-PhCl after 10 min of the reaction (X_10_) in the first and second runs (**B**); 4-PhCl conversion after 8 min (X_8_) in 10 consecutive cycles over FePd/ZS reduced with H_2_ in water at 30 °C (**C**). Reaction conditions: T = 30◦C, m_cat_ = 0.1 g, V_4-PhCl_ = 0.015 L, C_0 4-PhCl_ = 75 mg/L (0.8 mmol/L), pH = 7, V_H2_ = 0.6 L/h.

A convenient reduction method for Pd catalysts is their treatment with NaBH_4_ in water solution, which leads to H_2_ evolution and mild reduction of the catalyst. That is why the first catalytic experiments were performed using this reduction method. During this treatment H_2_ was continuously supplied. However, the expected blackening of the catalyst due to the metal oxides reduction was not observed for the monometallic catalysts after 1 h of the treatment. Only FePd/ZS changed its color to black. Nevertheless, all these catalysts were washed with distilled water from the sodium borohydride solution and tested in the conversion of 4-PhCl in water at 30 °C (Figure 8A).

All the catalysts were active in transformation of 4-PhCl (Figure 8A). The main product was phenol, and other unidentified products amounts were less than 1%. Most probably, they can contain cyclohexanol and/or cyclohexanone. The reaction rate depended on the composition of the catalyst. Fe/ZS showed the lowest activity: after the initial sharp drop of the 4-PhCl concentration the stable-state low reaction rate was observed during next 200 min. Pd/ZS was much more active, but the induction period of a low activity was observed during the first 20 min of the test. The bimetallic catalyst FePd/ZS provided a fast hydrodechlorination of 4-PhCl, and almost total conversion to phenol was achieved after 10 min.

After decantation of the reaction mixture the catalysts were tested in the second cycle using a fresh portion of 4-PhCl (Figure 8B). No additional reductive treatment was performed before second run. Fe/ZS (low) and FePd/ZS (high) showed respectively similar low and high 4-PhCl conversions in the first and second runs with only a slight improvement in the second run. However, X_10_ increased significantly for Pd/ZS. Five doses of 4-PhCl were successfully transformed to phenol over this catalyst (Supplementary Information, Figure S5) without loss of activity. The HDC of each 4-PhCl dose required only 10 min.

The increase in the activity in the second cycle and the sharp change in the reaction rate under the influence of the reaction mixture after 20 min of the reaction in the first run point to the possible reduction of palladium in Pd/ZS under the test conditions. It can be assumed that the catalyst reduction was caused by the appearance of the reaction product, phenol, in the reaction mixture, or by an increase in the catalyst’s ability to be reduced by hydrogen due to the action of the reaction medium. To test this assumption, we first tried to reduce the catalysts Pd/ZS and FePd/ZS in the form of a suspension in water with hydrogen at room temperature during 1 h.

This treatment was unsuccessful in the case of Pd/ZS. However, when Pd/ZS was treated with water solution of phenol in H_2_ flow, the catalyst turned black. This was an indication of the reduction of the palladium in the catalyst. After this treatment, a high conversion rate of 4-PhCl was achieved immediately without any induction period. Therefore, the treatment of Pd/ZS with aqueous phenol solution in the presence of H_2_ provided mild reduction at least part of PdO to Pd^0^ and was much more efficient than the treatment with sodium borohydride or with H_2_ in the absence of phenol. Note, that the treatment of Pd/ZS by phenol solution only, without H_2_ supply, provided no reductive action and no improvement in the catalytic efficiency.

In contrast, the reduction of FePd/ZS with H_2_ at 30 °C in water suspension proceeded successfully, the catalyst blackened fast and demonstrated the same activity in 4-PhCl transformation, as after treatment with NaBH_4_. Obviously, mild reduction with hydrogen at RT is a more environmentally friendly alternative to NaBH_4_ treatment since no additional products are formed except water.

The bimetallic catalyst was not only very active, but also highly stable. A catalyst batch of 0.1 g successfully converted 10 doses of 4-PhCl (see Figure 8C). The same results were obtained in the stability tests whatever the reduction method was used: with NaBH_4_ solution or only H_2_ at 30 °C (Figure 8C shows the data for the catalyst reduced with H_2_). A slight decrease in activity in the 10th cycle possibly resulted from the loss of catalyst during sampling. In each stability test, 150 mL (75 mg/L) of chlorophenol solution was successfully converted to phenol. Thus, FePd/ZS catalyst demonstrated a high HDC rate, excellent reducibility, and high stability in 4-PhCl HDC in water, which makes possible testing this catalyst in a flow-type reactor.

The possibility of the partial leaching of iron and/or palladium to the solution during reaction cannot be ruled out. To check this assumption, the reaction mixture after two consecutive runs of 4-PdCl HDC over the FePd/ZS catalyst was collected, separated from the solid catalyst by centrifugation and analyzed by the AAS method. The extremely low concentrations of Pd and Fe were detected in the reaction mixture. The degree of leaching was the same for both metals: 1.98% of the loading of each metal in the catalyst. These results confirm the predominantly heterogeneous nature of the HDC process.

No significant changes in textural properties of the catalysts after catalytic tests were found by N_2_ adsorption. Only a slight decrease in S_BET_ was observed for FePd/ZS (Table 1), while S_BET_ of Fe/ZS remained unchanged. Moreover, the average pore size and volume did not also change, confirming the stability of these catalysts under the reaction conditions.

Iron is hardly reducible at such mild conditions, and the absence of the reaction rate improvement for Fe/ZS under test conditions confirms this statement. The Fe/ZS catalyst demonstrated the same reaction rates after reductive treatment with NaBH_4_ and the aqueous solution of phenol, both in the presence of H_2_ (Figure S6 in Supplementary Material). No noticeable blackening of this catalyst was observed under both types of treatment.

Thus, the performance of the bimetallic FePd/ZS catalyst in 4-PhCl HDC is apparently much better than that of its monometallic counterparts, which suggests a synergistic effect of Fe and Pd. Considering the reduced Pd and oxidized Fe states in the tested catalysts, this effect can result from the close interaction between Pd^0^ and iron oxides in FePd/ZS detected by TPR-H_2_ and from the higher dispersion of Pd, which was proposed based on the XRD, TEM, and TPR-H_2_ results.

The combined action of phenol and hydrogen was shown to ensure successful reduction of Pd in Pd/ZS at room temperature (30 °C) in contrast to the treatment with sodium borohydride, pure hydrogen, or pure phenol. This fact requires additional study.

### 2.6. DRIFT Spectroscopy of CO Adsorbed on Pd/ZS after Reduction with Phenol and H_2_ in Water

**Figure 9:**
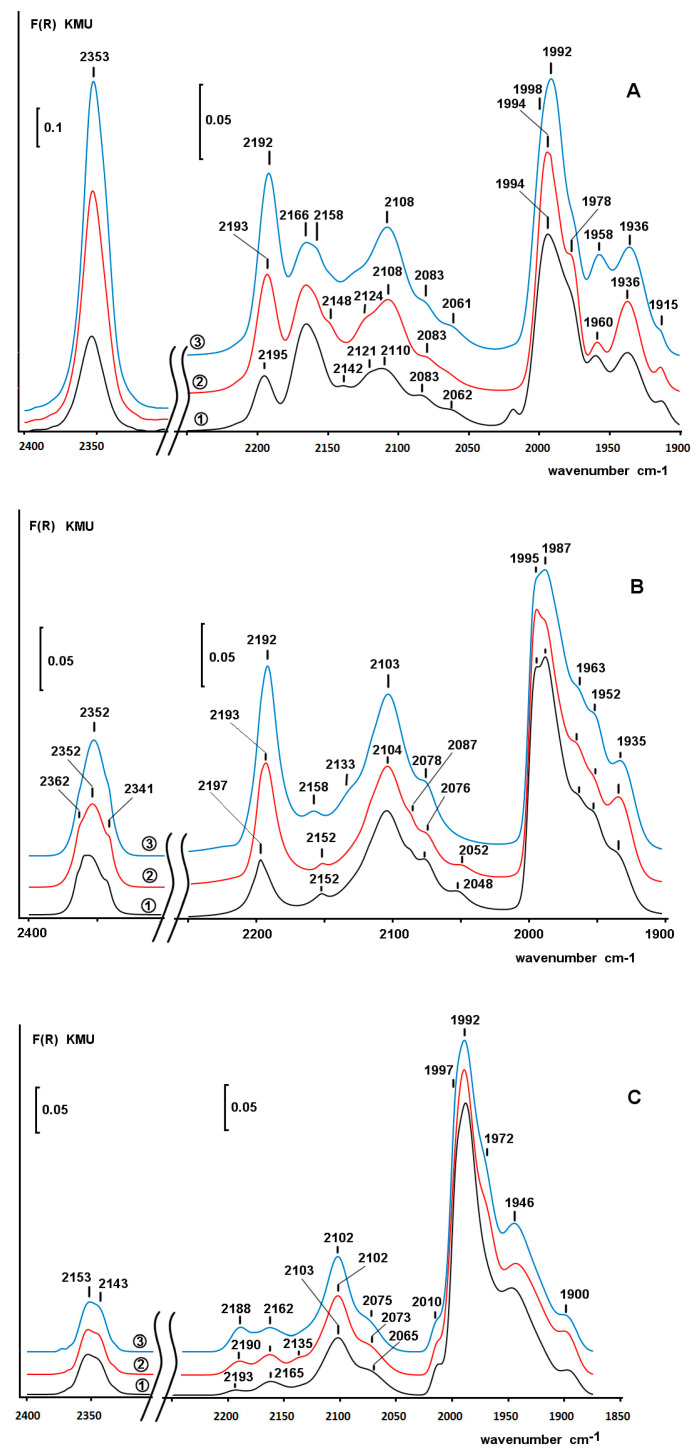
Difference IR spectra of CO adsorbed at RT on the Pd/ZS before (**A**), and after treatment with H_2_ + water solution of phenol (**B**); spectra of Pd/ZS after mild reduction with H_2_ at 200 °C (**C**). CO pressure: 5 Torr—curve 1; 20 Torr—curve 2; 50 Torr—curve 3. F(R)—Kubelka–Munk function (Kubelka–Munk units).

To study the catalyst evolution in the phenol–hydrogen mixture, a weighed amount of unreduced Pd/ZS catalyst was treated for 1 h with an aqueous solution of phenol in a stream of hydrogen. After treatment, the water was decanted, and the catalyst was carefully dried at RT in a stream of H_2_ and studied by DRIFT spectroscopy with CO adsorption (Figure 9B). For the comparison, the spectra of untreated catalyst (Figure 9A) and the same catalyst after mild (200 °C) dry reduction with H_2_ (Figure 9C) were also recorded. To prevent possible oxidation of the samples during a thermal vacuum treatment, the stage of a calcination in air was excluded after both types of the mild reduction (see Experimental section).

Spectrum of CO adsorbed at room temperature on the surface of the untreated Pd/ZS sample (Figure 9A) has a superposition of absorption bands at 2192–2195 cm^−1^ that corresponds to carbonyl complexes of Pd^2+^ cations (CO–Pd^2+^), and bands at 2142–2148, 2158, and 2166 cm^−1^ attributed to complexes of Pd^+^ cations (CO–Pd^+^). A weak band at 2121–2124 cm^−1^, in our opinion, can be also assigned to CO complexes of Pd^+^ species. The intense band at 2108–2110 cm^−1^ and the weak overlapping bands at 2061–2062 and 2083 cm^−1^ can be attributed to complexes of the metallic palladium (CO–Pd^0^).

In the frequency range below 2000 cm^−1^ the intense band at 1992–1994 cm^−1^ overlaps with less pronounced bands at 1958–1960 and 1936 cm^−1^. Poorly resolved weak bands at 1998, 1978, and 1915 cm^−1^ were observed as shoulders of the more intensive bands. All these bands can be attributed to CO complexes of the two palladium atoms (Pd^0^)_2_–CO, located in different coordination.

The intensive absorption band of CO_2_ coordinated to palladium cations was observed at about 2353 cm^−1^. Carbon dioxide can be produced via CO oxidation by surface oxygen.

In the spectrum of CO adsorbed on the surface of the Pd/ZS catalyst reduced in the presence of phenol and H_2_ (Figure 9B), the band of CO–Pd^2+^ complexes at 2192–2197 cm^−1^ remained intense, while the contribution from the absorption bands of CO–Pd^+^ complexes at 2152–2158 and 2133 cm^−1^ significantly decreased. On the contrary, the intensities of the absorption bands of CO–Pd^0^ complexes at 2103–2104, 2048–2052, 2076–2078, and 2087 cm^−1^ increased. Interestingly, for only a one band was observed for the unreduced catalysts at 2062 cm^−1^, while after reduction in H_2_+phenol two bands were detected at 2050 and 2077 cm^−1^. This indicates a change in the coordination environment of some palladium atoms upon reduction.

In the spectrum region below 2000 cm^−1^ of the reduced catalyst in addition to the superposition of bands corresponding to the CO complexes of the two palladium atoms (Pd^0^)_2_–CO, a noticeable contribution of a new absorption band appeared at 1987 cm^−1^. A new intense absorption band was also observed at 1952 cm^−1^ near the 1960 cm^−1^ band. This confirmed the hypothesis about the change in the coordination number of some palladium atoms on the surface upon reduction.

The amount of CO_2_ formed in this case was significantly lower because of the removal of oxygen from the surface during reduction. However, three overlapping bands observed at 2362, 2352, and 2341 cm^−1^ corresponded to CO_2_ coordinated to palladium cations located in different coordination environments.

Therefore, phenol+H_2_ treatment changed the coordination environment of some palladium atoms. The presence of phenol promoted the reduction of Pd^+^ to Pd^0^, but weakly affected the reduction of Pd^2+^ to Pd^+^.

Interestingly, the absence of the air calcination step increased the Pd^0^ concentration even though the temperature of thermal vacuum treatment was lower. Thus, after standard treatment at 550 °C the contribution from the superposition of bands positioned below 2000 cm^−1^ ((Pd^0^)_2_–CO complexes) was insignificant. The contribution from the bands corresponding to Pd^0^–CO complexes was also significantly lower than that of CO–Pd^+^ complexes at 2165 cm^−1^. However, after calcination at 400 °C without air exposure at high temperatures a significantly higher contribution from CO forms adsorbed on Pd^0^ was found as indicated by the bands at 2108–2110, 2161–2162, and 2183 cm^−1^, and the intense bands in the range of 1900–2000 cm^−1^.

Moreover, the spectra in Figure 5, Figure 6 and Figure 7 were recorded after severe reduction with H_2_ at 330 °C. For more correct comparison, we changed the conditions of the preliminary treatment of the fresh Pd/ZS to reduction with H_2_ at 200 °C without calcination. The spectra for this sample are shown in Figure 9C. They contain intense bands of CO–Pd^0^ complexes at 2102–2103 and 2065–2075 cm^−1^, weak bands of CO–Pd^+^ complexes at 2135 and 2162–2165 cm^−1^, the bands of CO–Pd^2+^ complexes at 2188–2193 cm^−1^. Consequently, under the treatment with H_2_ in the gas phase at 200 °C the reduction proceeded along the path Pd^2+^ → Pd^+^ → Pd^0^, that was, Pd^+^ accumulated first, and then reduced to Pd^0^. When the reduction was performed with H_2_ in the presence of phenol at RT in water solution, the accumulation of a partially reduced Pd^+^ did not occur because it was very quickly reduced to Pd^0^.

It seems that the partial reduction of Pd^2+^ to Pd^+^ by H_2_ opened a possibility for fast transformation of Pd^+^ to Pd^0^ under the influence of hydrogen formed during dissociative adsorption of phenol on the surface of catalyst, or by direct reduction with phenolate-ion adsorbed on the catalyst surface.

## 3. Discussion

Monometallic Pd/ZS and Fe/ZS and bimetallic FePd/ZS catalysts on a mesoporous support ZS were found to be active in the reductive dechlorination of 4-PhCl. The difference in physicochemical and catalytic properties between the monometallic Fe/ZS, Pd/ZS, and bimetallic FePd/ZS catalysts was well established. The Fe/ZS catalyst turned out to be the least active in this reaction. The Pd-containing mono- and bimetallic catalysts were much more efficient due to increased ability to reduce in mild conditions. A comparison of the TPR-H_2_ and the DRIFT spectroscopy results demonstrated that both mild (RT, H_2_, water, with or without phenol) and harsh (H_2_ at 320 °C for Pd-containing catalysts) treatments reduced at least part of Pd^2+^ to Pd^0^. Although the DRIFT spectroscopy demonstrated the presence of both Fe^0^ and oxidized iron species in the FePd/ZS after thermal-vacuum treatment, the oxidized forms partially covered the surface of the metal particles. Besides, these data were obtained after the harsh treatment: calcination at 550 °C followed by H_2_ reduction at 330 °C. Therefore, after the mild reduction treatment before the catalytic experiments, only the partial reduction of Fe (from Fe^3+^ to Fe^2+^) was expected in FePd/ZS and Fe/ZS. Therefore, the reduced catalysts most likely comprised Pd^0^, iron oxides (Fe_3_O_4_ and/or less probable FeO) on the mesoporous ZS support.

A strong interaction between Pd and iron oxide was highly probable in the bimetallic catalyst. TPR-H_2_ results confirmed this interaction. In the bimetallic sample compared to the monometallic ones the hydrogen evolution peak of the palladium hydride decomposition disappeared, and the iron reduction peak shifted to the much lower temperatures. The inhibition of PdH_x_ formation can be caused not only by the increase in the Pd dispersion, but also by the presence of iron, as it was observed in [66,67,68]. The reduction of the iron oxides can proceed at the lower temperatures under catalytic action of Pd, which was already in the Pd^0^ state when the iron oxides started reducing in the TPR-H_2_ conditions. Because of the strong interaction between the Pd and iron oxides the formation of Pd_x_Fe_y_O species was highly probable in the surface layer where palladium oxide contacts with the iron oxides. Earlier, we studied bimetallic iron-palladium catalysts on the carbon support by XPS and found that their structure was predominantly Pd/Fe_x_O_y_/C [20]. Such strong interaction causes heat adsorption changes for both reagent and hydrogen. Considering the strong interaction between Pd and iron oxides the following bimetallic catalyst structure can be proposed: Pd/PdxFeyO/Fe_x_O_y_/ZS. The highest probability of the strong interaction between Pd and iron oxides supported on SiO_2_ was earlier observed by TPR-H_2_ and XPS in the catalysts with the relatively high Fe content (more than 8 wt%) [21], similar to that used in our work. This may also affect the dispersion of the active phase. The high dispersion of the palladium particles on the surface of both palladium-containing catalysts can also be due to the presence of zirconium oxide in the support [53,54] and its mesoporous structure. However, even the small difference in the Pd particle sizes in mono- and bimetallic Pd-containing catalysts was an important factor, as HDC is often considered as a structure-sensitive reaction [20,69,70]. The presence of low-intensive broad Pd peak in the XDR profile of Pd/ZS and its absence for FePd/ZS confirmed the smaller Pd particle size in FePd/ZS. This was also indirectly confirmed by the TEM analysis. In about 30 TEM images of Pd/ZS the number of detected Pd particles was a bit higher than that in the images of FePd/ZS, possibly, due to a larger size of the Pd particles in the former catalysts. Thus, the strong interaction between Fe_x_O_y_ and Pd and higher dispersity of palladium in the bimetallic catalyst are the reasons for its improved catalytic performance.

The high selectivity to phenol with nearly no over-hydrogenation to cyclohexanol/cyclohexanon was the inherent property of all three studied catalysts. Fe in a PdFe catalyst prevented the over-hydrogenation of phenol observed on the monometallic Pd catalyst [21]. However, in our work, we did not observe the significant difference in selectivity to phenol between monometallic and bimetallic catalysts, only minor amounts of side products were formed over all three catalysts.

The high stability of the Pd-containing catalysts was demonstrated in the 10 consecutive cycles for FePd/ZS and five cycles for Pd/ZS. The stability of the catalysts was confirmed by the absence of significant changes in their texture after catalytic tests according to the N_2_ physisorption data, and by low leaching of metals into the reaction medium, according to the AAS results. This is especially important for the industrial use in water decontamination. Apparently, the high stability was at least partly due to the stability of the carrier in an aqueous medium and the strong bond of the active components with its surface. Our work opens the way for the development of a flow-type reactor for the hydrodechlorination of 4-PhCl or other chlorinated molecules.

As for Fe/ZS catalyst, it demonstrated lower efficiency in hydrogenation than the other two samples. The reaction rate over this catalyst decreased after first 10 min of the test, possibly due to the iron chloride formation under the influence of side product of HDC (HCl), and then the conversion of 4-PhCl to phenol continued at a constant, albeit at a low rate.

The great advantage of the bimetallic FePd/ZS catalyst was its improved reducibility at mild conditions. This catalyst can be effectively reduced in less than 1 h at 30 °C in an aqueous suspension not only with NaBH_4_ solution, but even with H_2_. This is a significant advantage because it eliminates the additional reducing agent (e.g., sodium borohydride) and high temperatures, making the reduction process less energy demanding and more environmentally friendly.

In contrast, Pd/ZS has worse reducibility at mild conditions. The treatment with an aqueous solution of sodium borohydride for 1 h both without and with additional supply of H_2_ did not lead to any noticeable blackening of the catalyst. Moreover, a noticeable induction period with a low rate of 4-PhCl transformation was observed in the kinetic curve (Figure 8A). However, after this period, the reaction rate increased noticeably, and the catalyst simultaneously turned black. Therefore, we can suggest that phenol as a reaction product can cause these changes. There are two possible reasons for this effect: participation of phenol in the hydrodechlorination of 4-PhCl and its reductive effect on the catalyst.

The increase in the reaction rate due to the addition of 5% isopropanol to the solvent water was observed under HDC of 4-PhCl over Pd/C catalyst [71]. Isopropanol can serve as an additional source of hydrogen in the reaction. Replacement of chlorine in chlorinated organic compounds can be performed using isopropanol as a single source of hydrogen [72,73]. In the presence of noble metals much milder conditions can be used [73,74,75] than in the case of Raney nickel [72]. However, to the best of our knowledge, phenol has never been mentioned in the literature as a hydrogen donor in the HDC reaction.

In contrast, the participation of phenol in the catalyst reduction was successfully confirmed in the direct experiment: Pd/ZS catalyst was reduced by the treatment with an aqueous solution of phenol in the presence of hydrogen. The ability of a palladium catalyst to be reduced under the combined action of an aqueous solution of phenol and hydrogen is a remarkably interesting result of our work.

**Scheme 1:**
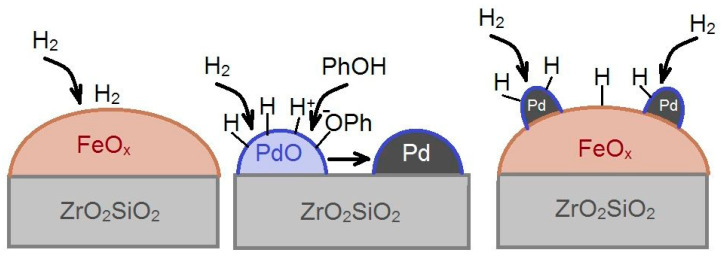
A proposed structure of the catalysts and their interaction with H_2_ and phenol.

The DRIFT spectroscopy demonstrated that the combined action of phenol and H_2_ at RT promoted the reduction of Pd^+^ to Pd^0^, but weakly affected the reduction of Pd^2+^ to Pd^+^. The increase in the polarity of the O−H bond under the action of the benzene ring leads to the dissociation of phenol in aqueous solutions with the formation of a phenolate ion. For this reason, the redox potential of phenol is pH dependent (E_red_^0^ = 0.86 V vs. NHE for pH = 12) [76]. Besides, proton can be eliminated because of the dissociative adsorption of phenol on the catalyst surface, which produces phenolate ion on the catalyst surface. Due to strong adsorption ability of phenol the surface of the catalyst will retain a significant amount of the phenol formed in HDC or phenolate ions formed under its dissociation. Phenolate ion has a noticeable reductive ability. It seems that partial reduction of palladium Pd^2+^ to Pd^+^ started under the influence of H_2_ at mild condition, which was observed in the TPR-H_2_ data. As soon as partial reduction of Pd^2+^ with H_2_ began in water medium, the further reduction of Pd^+^ to Pd^0^ can occur under the action of H_2_. The presence of the phenolate ion on the catalyst surface strongly contributed to this process and a noticeable reduction of the surface layer of the catalyst. The possible structures of the Pd/ZS and FePd/ZS catalysts and possible mechanisms of their reduction under mild conditions are illustrated in Scheme 1.

The reduction of metal oxides in a catalyst under the action of alcohols, including cyclohexanol, was earlier noted. For example, phenols were used as reductants for MnO_2_ [77].

Several alcohols were used as hydrogen donors for catalytic transfer hydrogenation of methyl levulinate to γ-valerolacton [78]. XRD study of the used catalysts showed that alcohols, and especially secondary alcohols, are the excellent reducing agents not only for methyl levulinate, but also for supported metal oxides (CuO, NiO) (the concept of “simultaneously activated catalysts”). Interestingly, among other secondary alcohols, the authors of the work [78] tested cyclohexanol that is the hydrogenated derivative of phenol. Cyclohexanol is a poor reducing agent for methyl levulinate, but it is highly effective in reducing metals (Cu and Ni) in oxides. PdO is obviously more easily reducible than CuO and NiO.

Oxidation of isopropanol on the surface of a wide range of metal oxides was earlier studied [79]. The redox interaction of Fe_2_O_3_ with isopropanol to form isopropoxide species and OH-groups was observed at 231 °C, but PdO interacted with this alcohol at much lower temperature of 64 °C. No signs of metal oxide reduction were found by the XPS, which was expectable because isopropanol was oxidized in the presence of O_2_.

The presented results indicate that close interaction of palladium with iron oxide which occurs on the surface of the mesoporous support ZS provides an increase in the dispersion of palladium in the bimetallic catalyst and its ability to be reduced in mild conditions. That is why the bimetallic FePd/ZS catalyst demonstrated the excellent catalytic performance in HDC of 4-PhCl to phenol in aqueous medium under mild conditions.

## 4. Materials and Methods

### 4.1. Synthesis of the ZS Support

The aqueous solution of ZrO(NO_3_)_2_∙H_2_O (99.5%, Acros Organics, Geel, Belgium) was added dropwise at RT at continuous stirring to the CTAB aqueous solution. After 2 h of stirring the solution was aged for 28 h at 95 °C. A bright orange product was dried for 6 days at RT, and then suspended in water. Meanwhile tetraethyl orthosilicate (TEOS, Si(OC_2_H_5_)_4_) (99%, BioChemica, Billingham, UK) was added to 1M solution of tetramethylammonium hydroxide ((CH_3_)_4_NOH) (Acros Organics, Geel, Belgium) in distilled water; the prepared solution was slowly dripped to the suspension, and stirred for 1.5 h. The produced white gel material was aged for 48 h at 90 °C, dried for 3 h at 200 °C to form porous orange powder. The powder was heated in air (2 °C/min) up to 500 °C and calcined at this temperature for 4 h, then heated up to 550 °C and calcined for 3.5 h. As a result, a white powder support with the Si:Zr atomic ratio of 1.2 was synthesized.

### 4.2. Synthesis of the Catalysts

The catalysts were synthesized by the wet impregnation of the ZS support with Fe(NO_3_)_3_ × 9 H_2_O (chemically pure, Merck, Madison, NY, USA) and Pd(NO_3_)_2_ (pure, Aurat, Moscow, Russia) water solutions. Initially the support was dried at 150 °C for 1 h, moistened with a minimum amount of distilled water and the salt solution in the minimum amount of water was added dropwise at continuous stirring. After 2 h stirring the water was evaporated in a water bath (approx. 1.5 h), and the material was dried in air at RT for one night, at 150 °C for 1 h and calcined at 400 °C (heating at 1.6 °C/min, isothermal calcination for 5 h). Bimetallic catalyst was prepared by the simultaneous impregnation with both salts. The synthesized Pd/ZrO_2_SiO_2_, Fe/ZrO_2_SiO_2_, and FePd/ZrO_2_SiO_2_ catalysts were named as Pd/ZS, Fe/ZS, and FePd/ZS.

### 4.3. Catalytic Tests

The catalysts were tested at 30 °C and pH = 7 in a 30 mL three-necked reactor with the thermostatically controlled jacket. One neck was connected to a reflux condenser, the second one to H_2_ supply (10 mL/min, 0.045 mmol/min), and the third neck was closed with a rubber septum. 0.1 g of the unreduced catalyst was placed into the reactor. It was reduced at RT by one of three methods: (i) with 20 mL of aqueous NaBH_4_ (p.a., Sigma-Aldrich, St. Louis, MO, USA)(0.1 mol/L) with simultaneous H_2_ supply (10 mL/min, 0.045 mmol/min); (ii) by H_2_ treatment (10 mL/min, 0.045 mmol/min) of the catalyst in water (15 mL); or (iii) by treatment with 15 mL of water solution of phenol (75 mg/L, 0.8 mmol/L) with the simultaneous H_2_ supply (10 mL/min, 0.045 mmol/min).

After the treatment with sodium borohydride or phenol solutions, the reducing solution was removed by decantation and the catalyst was carefully washed with 4 × 20 mL of distilled water. Then an aqueous solution of 4-PhCl was added and the reaction was initiated by supplying of H_2_. 15 mL of 4-chlorophenol (4-PhCl) (99+%, Lancaster, Germany) solution in distilled water (75 mg/L, 0.588 mmol/L) was used in each catalytic ran.

Samples for analysis were taken with a syringe through the septum at regular intervals and centrifuged to separate small catalyst particles.

The conversion of 4-PhCl was calculated according to Equation (1)

(1) $$X_{t}=\frac{C_{0}-C_{t}}{C_{0}}$$

where *C*_0_ is the concentration of 4-PhCl (mmol/L) in the initial solution, and *C_t_* is its concentration after *t* min of reaction.

To check the stability of the catalysts, after the complete 4-PhCl conversion was achieved, the reaction solution was removed by decantation after sedimentation of the catalyst, and the new portion of 15 mL 4-PhCl aqueous solution was added. Up to 10 cycles were performed for Pd-containing catalysts.

The products were analyzed by HPLC on an Agilent 1100 Series (Agilent Technologies Inc., Colorado Springs, CO, USA) instrument using a Zorbax SB-C18 column (15 cm) and a UV-detector at 278 nm. The column was maintained at 35 °C. Mobile phase A consisted of deionized water with 0.1 M/L formic acid. Mobile phase B consisted of acetonitrile. The A:B ratio of 55:45 vol% at a flow rate of 1.0 mL·min^−1^ was utilized. The full method time was 7 min. The injection volume was 20 μL for each sample.

Calibration was performed using five 4-PhCl aqueous solutions, prepared from stock solution (150 mg/L) by three two-fold dilutions.

The results of the HPLC analysis and calibration curves were used to plot kinetic curves.

### 4.4. Characterization of the Catalysts

Low temperature nitrogen physisorption isotherms were recorded on an Autosorb-1 analyzer (Quantachrome Instruments, Boynton Beach, FL, USA) after degassing samples in vacuum at 200 °C for 3 h. The specific surface area (S_BET_) was calculated using the multi-point BET method, while the pore volume and the average pore diameter were obtained from the pore size distribution calculated by the Barrett–Joyner–Halenda method (BJH) using the desorption branch of isotherm.

X-ray powder diffraction patterns were recorded on a MiniFlex 300/600 (Rigaku, Tokyo, Japan) diffractometer (CuKα radiation, 1.5418 Å). The XRD patterns were collected in the 2θ range of 5–80° with a step size of 0.02°. The ICDD powder diffraction files were used for the phase identification.

Temperature-programmed reduction with H_2_ (TPR-H_2_) was performed by heating unreduced catalyst (approx. 50 mg) from 30 to 900 °C (10 °C/min) in the mixture of 5% H_2_–95 %Ar (30 mL/min) on an USGA-101 apparatus (Unisit, Moscow, Russia). Before analysis, the sample was treated in Ar flow for 30 min at 300 °C and cooled to 30 °C. A thermal conductivity detector (TCD) at 60 °C was used to monitor the changes in the gas mixture composition. The TCD signal was calibrated using NiO reduction.

Scanning electron microscopy (SEM) images were recorded on a JEOL JSM–6390 microscope (JEOL Ltd., Tokyo, Japan), equipped with an energy-dispersive spectroscopy (EDS) accessory. The sample was fixed on a copper-zinc table using a double-sided conductive carbon tape. Accelerating voltage of 10 kV and working distance of 8–10 mm were used. The EDX spectra and elemental maps were recorded at accelerating voltage of 15 kV.

The high-resolution transmission electron microscopy (HRTEM) images were obtained at accelerating voltage of 200 kV on a JEM 2100F-UHR instrument (JEOL, Tokyo, Japan) equipped an EDS accessory.

Atomic-adsorption analysis (AAS) was performed on an iCE 3000 Series AA instrument (Thermo Scientific, Waltham, MA, USA), using flame atomization (acetylene-air flame). The data were processed using the Thermo Scientific SOLAAR software (version 1.30). To convert metals into a solution, weighed portions of the catalysts were dissolved in a small amount of aqua regia with stirring and heating for 2 h. The resulting solution was left overnight and then diluted with distilled water to a metal content of about 4 mg/L. Just before analysis the instrument was calibrated using freshly prepared standard solutions of palladium and Fe^3+^ nitrates.

Diffusion reflection Fourier-transformed IR-spectra (DRIFT) were registered using an EQUINOX 55/S spectrometer (Bruker, Ettlinger, Germany) in the wavenumber range of 4000–800 cm^−1^ with a resolution of 4 cm^−1^ and averaging over 1024 scans. The powder of the unreduced catalyst was placed in a quartz ampule with the CaF_2_ window, calcined at 550 °C for 1 h in air and 2 h in a vacuum of better than 5 × 10^−5^ Torr. The differential spectra of adsorbed CO were obtained by subtracting the background spectrum from the experimental spectrum recorded after CO adsorption, followed by the baseline correction using the OPUS software version 6.0 (Bruker). The samples were also investigated after in situ reduction in H_2_ atmosphere at 330 °C and 180 Torr.

The Pd/ZS sample was also investigated after reductive treatments at milder conditions: (1) Reduction with H_2_ at 200 °C was the first type of milder treatments. (2) The separate portion of Pd/ZS was simultaneously treated with 15 mL of water solution of phenol (75 mg/L) and H_2_ (10 mL/min, 0.045 mmol/min) at 30 °C for 1 h. The stage of calcination in air was excluded after both types of mild reduction. The temperature was raised stepwise from 30 to 400 °C with a simultaneous decrease in air pressure in ampoules containing the weighed amounts of this sample before and after treatment. The samples were heated to 400 °C and kept at this temperature for 2 h in a vacuum better than 5 × 10^−5^ Torr before CO adsorption experiment was started.

CO and H_2_ were purified by passing through a liquid nitrogen trap and holding for a long time over the calcined zeolite.

## 5. Conclusions

The composition of the support, including silicon and zirconium oxides, as well as its mesoporous structure with a predominant pore size of about 4 nm, provide a high dispersion of palladium and iron oxides in the composition of the mono- and bimetallic catalysts under study. The uniform distribution of the components on the surface of this support found by SEM-EDS leaded to a close interaction between palladium and iron oxides, which was confirmed by TPR-H_2_ and DRIFT spectroscopy of adsorbed CO both after harsh and mild reduction. For these reasons, the bimetallic FePd/ZS catalyst demonstrated much higher reducibility compared to the monometallic Pd/ZS and Fe/ZS. FePd/ZS can be easily reduced at 30 °C in water by H_2_ treatment. As a result, this catalyst demonstrated the excellent activity, selectivity to phenol and stability in HDC of 4-PhCl in aqueous solution under the action of H_2_ at mild conditions. The Pd/ZS was less reducible, and therefore much less active than FePd/ZS in the first run after reduction with sodium borohydride. However, under the influence of phenol in the presence of H_2_ PdO in Pd/ZS can be efficiently reduced both at reaction conditions and by pre-treatment before the catalytic test. This pre-treatment significantly increases the reaction rate, and after the proper reduction Pd/ZS also showed high activity and stability in 4-PhCl transformation to phenol. The DRIFT spectroscopy of CO adsorbed on FePd/ZS reduced in harsh (H_2_, 330 °C), medium (H_2_, 200 °C) and mild conditions (H_2_ + aqueous solution of phenol) allowed identifying the reasons for the reducing action of phenol solution. It provided the fast transformation of Pd^+^ to Pd^0^.

The obtained results demonstrate the possibility of the fast, efficient, and selective utilization of chlorophenol to phenol on the bimetallic palladium catalyst, prepared on the mesoporous ZS support and reduced under mild conditions with hydrogen at room temperature in an aqueous medium, which is an environmentally friendly and energy-saving method. A monometallic Pd/ZS can be efficient also in this reaction after proper reductive treatment. The complete conversion of 4-PhCl to phenol proceeded rapidly. The results obtained open up possibilities for using other mesoporous carriers for the synthesis of effective catalysts for the hydrodechlorination of hazardous environmental pollutants. Natural porous materials, for example, chitosan or its derivatives have good prospects. In our future work, we propose to obtain catalysts using such materials as a carrier, since they are stable in aqueous solutions and can provide interesting catalytic properties of active metals attached to them. It is also interesting to study the possibilities of the obtained systems in the processing of other environmental toxicants of a similar structure, for example, polychlorinated phenols. They can also be active in the conversion of chlorinated components of medicines. Water pollution by chlorinated medicines and their metabolic products is a pressing environmental problem and using the catalysts on ZS mesoporous oxide support described in the article could provide a solution for this problem.

## Figures and Tables

**Table 1:** Morphological properties of unreduced catalysts (fresh) and the same systems after catalytic tests.

Sample	S_BET_ (m^2^/g)		V_pore_ (cm^3^/g)		Average Pore Width (nm)	
	Fresh	After Catalytic Test	Fresh	After Catalytic Test	Fresh	After Catalytic Test
Pd/ZS	339 ± 34	N/D *	0.374	N/D	3.7	N/D
Fe/ZS	331 ± 33	320 ± 32	0.266	0.297	4.1	4–5
FePd/ZS	364 ± 36	300 ± 30	0.292	0.303	4.1	4–5
ZS	529 ± 53	-	0.397	-	3.7	-

* This catalyst has not been studied after catalytic tests.

**Table 2:** Metal content and surface composition of the unreduced catalysts.

Catalyst	Metal Content, wt% (AAS Data)		Composition, at % (SEM-EDS Data)				
	Pd	Fe	O	Si	Zr	Pd	Fe
Pd/ZS	0.86 ± 0.02	–	79.3	18.9	1.7	0.1	–
Fe/ZS	–	9.6 ± 0.2	80.1	15.6	1.6	–	2.7
FePd/ZS	0.89 ± 0.01	8.2 ± 0.4	77.9	18.1	1.7	0.1	2.2

## Data Availability

The data presented in this study are available on request from the corresponding author. They are not contained in any publicly available database.

## Supplementary Materials

The following are available online, Figure S1. SEM images of Pd/ZS and Fe/ZS (after reduction with H_2_ at 320 and 500 °C, respectively); the maps of elements distribution on the surface produced by EDS. Figure S2. HR TEM images for Pd/ZS after the simultaneous treatment with water solution of phenol (75 mg/L) and H_2_ (10 mL/min) at 30 °C (1 h). Figure S3. HR TEM images for FePd/ZS after the treatment with H_2_ (10 mL/min) in water at 30 °C (1 h). Figure S4. HR TEM images for Fe/ZS after the simultaneous treatment with water solution of phenol (75 mg/L) and H_2_ (10 mL/min) at 30 °C (1 h). Figure S5. The conversion of 4-PhCl after 8 min of reaction on Pd/ZS in five consecutive runs. The preliminary reduction before the first run was performed using aqueous solution of NaBH_4_ and H_2_. Figure S6. A comparison of Fe/ZS catalytic performance after reduction with aqueous solution of NaBH_4_ and H_2_ + phenol in aqueous solution, both treatments were performed at RT, 1 h.

## Sample Availability

Samples of the compounds are not available from the authors.

## References

[1] Lokteva E., Golubina E., Likholobov V., Lunin V., Tundo P., He L.-N., Lokteva E., Mota C. (2016). Disposal of Chlorine-Containing Wastes. Chemistry Beyond Chlorine.

[2] Shi W., Yu N., Jiang X., Han Z., Wang S., Zhang X., Wei S., Giesy J.P., Yu H. (2017). Influence of blooms of phytoplankton on concentrations of hydrophobic organic chemicals in sediments and snails in a hyper-eutrophic, freshwater lake. Water Res..

[3] Ma J., Hung H., Tian C., Kallenborn R. (2011). Revolatilization of persistent organic pollutants in the Arctic induced by climate change. Nat. Clim. Chang..

[4] Juhler R.K., Sørensen S.R., Larsen L. (2001). Analysing transformation products of herbicide residues in environmental samples. Water Res..

[5] Yuan G., Keane M.A. (2003). Liquid phase catalytic hydrodechlorination of chlorophenols at 273 K. Catal. Commun..

[6] Tundo P., Perosa A., Selva M., Zinovyev S.S. (2001). A mild catalytic detoxification method for PCDDs and PCDFs. Appl. Catal. B Environ..

[7] Simagina V.I., Netskina O.V., Tayban E.S., Komova O.V., Grayfer E.D., Ischenko A.V., Pazhetnov E.M. (2010). The effect of support properties on the activity of Pd/C catalysts in the liquid-phase hydrodechlorination of chlorobenzene. Appl. Catal. A Gen..

[8] Gómez-Quero S., Cárdenas-Lizana F., Keane M.A. (2011). Liquid phase catalytic hydrodechlorination of 2,4-dichlorophenol over Pd/Al_2_O_3_: Batch vs. continuous operation. Chem. Eng. J..

[9] Wu Y., Gan L., Zhang S., Song H., Lu C., Li W., Wang Z., Jiang B., Li A. (2018). Carbon-nanotube-doped Pd-Ni bimetallic three-dimensional electrode for electrocatalytic hydrodechlorination of 4-chlorophenol: Enhanced activity and stability. J. Hazard. Mater..

[10] Cheng L., Jin Z., Wang X. (2013). Hydrodechlorination of chlorophenols at low temperature over highly defective Pd catalyst. Catal. Commun..

[11] Raut S.S., Shetty R., Raju N.M., Kamble S.P., Kulkarni P.S. (2020). Screening of zero valent mono/bimetallic catalysts and recommendation of Raney Ni (without reducing agent) for dechlorination of 4-chlorophenol. Chemosphere.

[12] Ma X., Liu S., Liu Y., Li X., Li Q., Gu G., Xia C. (2020). Promoted liquid-phase hydrodechlorination of chlorophenol over Raney Ni via controlling base: Performance, mechanism, and application. Chemosphere.

[13] Liu M., Huang R., Li C., Che M., Su R., Li S., Yu J., Qi W., He Z. (2019). Continuous rapid dechlorination of p-chlorophenol by Fe-Pd nanoparticles promoted by procyanidin. Chem. Eng. Sci..

[14] Han B., Liu W., Li J., Wang J., Zhao D., Xu R., Lin Z. (2017). Catalytic hydrodechlorination of triclosan using a new class of anion-exchange-resin supported palladium catalysts. Water Res..

[15] Nieto-Sandoval J., Munoz M., de Pedro Z.M., Casas J.A. (2018). Fast degradation of diclofenac by catalytic hydrodechlorination. Chemosphere.

[16] Agency for Toxic Substances and Disease Registry (ATSDR) (1998). Toxicological Pro-file for Phenol (Update).

[17] Diaz E., Mohedano A.F., Casas J.A., Shalaby C., Eser S., Rodriguez J.J. (2016). On the performance of Pd and Rh catalysts over different supports in the hydrodechlorination of the MCPA herbicide. Appl. Catal. B Environ..

[18] Hashimoto Y., Uemichi Y., Ayame A. (2005). Low-temperature hydrodechlorination mechanism of chlorobenzenes over platinum-supported and palladium-supported alumina catalysts. Appl. Catal. A Gen..

[19] Klokov S.V., Lokteva E.S., Golubina E.V., Maslakov K.I., Levanov A.V., Chernyak S.A., Likholobov V.A. (2016). Effective Pd/C catalyst for chlorobenzene and hexachlorobenzene hydrodechlorination by direct pyrolysis of sawdust impregnated with palladium nitrate. Catal. Commun..

[20] Golubina E.V., Lokteva E.S., Lunin V.V., Telegina N.S., Stakheev A.Y., Tundo P. (2006). The role of Fe addition on the activity of Pd-containing catalysts in multiphase hydrodechlorination. Appl. Catal. A Gen..

[21] Witońska I.A., Walock M.J., Binczarski M., Lesiak M., Stanishevsky A.V., Karski S. (2014). Pd–Fe/SiO_2_ and Pd–Fe/Al_2_O_3_ catalysts for selective hydrodechlorination of 2,4-dichlorophenol into phenol. J. Mol. Catal. A Chem..

[22] Jin Z., Yu C., Wang X., Wan Y., Li D., Lu G. (2011). Liquid phase hydrodechlorination of chlorophenols at lower temperature on a novel Pd catalyst. J. Hazard. Mater..

[23] de Pedro Z.M., Diaz E., Mohedano A.F., Casas J.A., Rodriguez J.J. (2011). Compared activity and stability of Pd/Al_2_O_3_ and Pd/AC catalysts in 4-chlorophenol hydrodechlorination in different pH media. Appl. Catal. B Environ..

[24] Shao Y., Xu Z., Wan H., Wan Y., Chen H., Zheng S., Zhu D. (2011). Enhanced liquid phase catalytic hydrodechlorination of 2,4-dichlorophenol over mesoporous carbon supported Pd catalysts. Catal. Commun..

[25] Yuan G., Keane M.A. (2004). Liquid phase hydrodechlorination of chlorophenols over Pd/C and Pd/Al_2_O_3_: A consideration of HCl/catalyst interactions and solution pH effects. Appl. Catal. B Environ..

[26] Yuan G., Keane M.A. (2003). Catalyst deactivation during the liquid phase hydrodechlorination of 2,4-dichlorophenol over supported Pd: Influence of the support. Catal. Today.

[27] Yuan G., Keane M.A. (2004). Role of base addition in the liquid-phase hydrodechlorination of 2,4-dichlorophenol over Pd/Al_2_O_3_ and Pd/C. J. Catal..

[28] Xiong J., Ma Y., Yang W., Zhong L. (2018). Rapid, highly efficient and stable catalytic hydrodechlorination of chlorophenols over novel Pd/CNTs-Ni foam composite catalyst in continuous-flow. J. Hazard. Mater..

[29] Ding X., Yao Z., Xu Y., Liu B., Liu Q., She Y. (2018). Aqueous-phase hydrodechlorination of 4-chlorophenol on palladium nanocrystals: Identifying the catalytic sites and unraveling the reaction mechanism. J. Catal..

[30] Jadbabaei N., Ye T., Shuai D., Zhang H. (2017). Development of palladium-resin composites for catalytic hydrodechlorination of 4-chlorophenol. Appl. Catal. B Environ..

[31] Molina C.B., Pizarro A.H., Casas J.A., Rodriguez J.J. (2014). Aqueous-phase hydrodechlorination of chlorophenols with pillared clays-supported Pt, Pd and Rh catalysts. Appl. Catal. B Environ..

[32] Fan M., Long Y., Zhu Y., Hu X., Dong Z. (2018). Two-dimensional covalent-organic-framework-derived nitroen-rich carbon nanosheets modified with small Pd nanoparticles for the hydrodechlorination of chlorophenols and hydrogenation of phenol. Appl. Catal. A Gen..

[33] Deng H., Fan G., Wang C., Zhang L. (2014). Aqueous phase catalytic hydrodechlorination of 4-chlorophenol over palladium deposited on reduced graphene oxide. Catal. Commun..

[34] Choi M., Kleitz F., Liu D., Lee H.Y., Ahn W.-S., Ryoo R. (2005). Controlled Polymerization in Mesoporous Silica toward the Design of Organic−Inorganic Composite Nanoporous Materials. J. Am. Chem. Soc..

[35] Liu S.G., Wang H., Li J.P., Zhao N., Wei W., Sun Y.H. (2007). A facile route to synthesize mesoporous zirconia with ultra high thermal stability. Mater. Res. Bull..

[36] Li R., Yu F., Li F., Zhou M., Xu B., Xie K. (2009). One-pot synthesis of superacid catalytic material SO_4_^2−^/ZrO_2−_SiO_2_ with thermostable well-ordered mesoporous structure. J. Solid State Chem..

[37] Bacani R., Martins T.S., Fantini M.C.A., Lamas D.G. (2016). Structural studies of mesoporous ZrO_2−_CeO_2_ and ZrO_2−_CeO_2_/SiO_2_ mixed oxides for catalytical applications. J. Alloy. Compd..

[38] Chen G., Lei T., Wang Z., Liu S., He X., Guan Q., Xin X., Xu H. (2019). Preparation of higher alcohols by biomass-based syngas from wheat straw over CoCuK/ZrO_2−_SiO_2_ catalyst. Ind. Crop. Prod..

[39] Liu W., Li L., Zhang X., Wang Z., Wang X., Peng H. (2018). Design of Ni-ZrO_2_@SiO_2_ catalyst with ultra-high sintering and coking resistance for dry reforming of methane to prepare syngas. J. CO_2_ Util..

[40] Thommes M., Kaneko K., Neimark A.V., Olivier J.P., Rodriguez-Reinoso F., Rouquerol J., Sing K.S.W. (2015). Physisorption of gases, with special reference to the evaluation of surface area and pore size distribution (IUPAC Technical Report). Pure Appl. Chem..

[41] Sharma M., Jain P., Mishra A., Mehta A., Choudhury D., Hazra S., Basu S. (2017). Variation of surface area of silica monoliths by controlling ionic character/chain length of surfactants and polymers. Mater. Lett..

[42] Christy E.J.S., Alagar R., Anitha Pius D.M. (2020). Porous nonhierarchical CeO_2_/SiO_2_ monolith for effective degradation of organic pollutants. Environ. Nanotechnol. Monit. Manag..

[43] Valenzuela M.A., Bosch P., Jiménez-Becerrill J., Quiroz O., Páez A.I. (2002). Preparation, characterization and photocatalytic activity of ZnO, Fe_2_O_3_ and ZnFe_2_O_4_. J. Photochem. Photobiol. A Chem..

[44] Armendariz H., Aguilar-Rios G., Salas P., Valenzuela M.A., Schifter I., Arriola H., Nava N. (1992). Oxidative dehydrogenation of n-butane on iron-zinc oxide catalysts. Appl. Catal. A Gen..

[45] Sepúlveda J.H., Fígoli N.S. (1993). The influence of calcination temperature on Pd dispersion and hydrogen solubility in Pd/SiO_2_. Appl. Surf. Sci..

[46] Boudart M., Hwang H.S. (1975). Solubility of hydrogen in small particles of palladium. J. Catal..

[47] Matam S.K., Otal E.H., Aguirre M.H., Winkler A., Ulrich A., Rentsch D., Weidenkaff A., Ferri D. (2012). Thermal and chemical aging of model three-way catalyst Pd/Al_2_O_3_ and its impact on the conversion of CNG vehicle exhaust. Catal. Today.

[48] Niu L., Liu X., Liu X., Lv Z., Zhang C., Wen X., Yang Y., Li Y., Xu J. (2017). In Situ XRD Study on Promotional Effect of Potassium on Carburization of Spray-dried Precipitated Fe_2_O_3_ Catalysts. ChemCatChem.

[49] Wendel J., Manchili S.K., Hryha E., Nyborg L. (2020). Reduction of surface oxide layers on water-atomized iron and steel powder in hydrogen: Effect of alloying elements and initial powder state. Thermochim. Acta.

[50] Wan H.-J., Wu B.-S., Zhang C.-H., Xiang H.-W., Li Y.-W., Xu B.-F., Yi F. (2007). Study on Fe–Al_2_O_3_ interaction over precipitated iron catalyst for Fischer–Tropsch synthesis. Catal. Commun..

[51] Nielsen M.R., Moss A.B., Bjørnlund A.S., Liu X., Knop-Gericke A., Klyushin A.Y., Grunwaldt J.-D., Sheppard T.L., Doronkin D.E., Zimina A. (2020). Reduction and carburization of iron oxides for Fischer–Tropsch synthesis. J. Energy Chem..

[52] Urasaki K., Tanimoto N., Hayashi T., Sekine Y., Kikuchi E., Matsukata M. (2005). Hydrogen production via steam–iron reaction using iron oxide modified with very small amounts of palladium and zirconia. Appl. Catal. A Gen..

[53] Narui K., Furuta K., Yata H., Nishida A., Kohtoku Y., Matsuzaki T. (1998). Catalytic activity of PdO/ZrO_2_ catalyst for methane combustion. Catal. Today.

[54] Shi C.-K., Yang L.-F., Wang Z.-C., He X.-E., Cai J.-X., Li G., Wang X.-S. (2003). Promotion effects of ZrO_2_ on the Pd/HZSM-5 catalyst for low-temperature catalytic combustion of methane. Appl. Catal. A Gen..

[55] Hadjiivanov K.I., Vayssilov G.N. (2002). Characterization of oxide surfaces and zeolites by carbon monoxide as an IR probe molecule. Advances in Catalysis.

[56] Bertarione S., Scarano D., Zecchina A., Johánek V., Hoffmann J., Schauermann S., Frank M.M., Libuda J., Rupprechter G., Freund H.-J. (2004). Surface Reactivity of Pd Nanoparticles Supported on Polycrystalline Substrates as Compared to Thin Film Model Catalysts:  Infrared Study of CO Adsorption. J. Phys. Chem. B.

[57] Lear T., Marshall R., Antonio Lopez-Sanchez J., Jackson S.D., Klapötke T.M., Bäumer M., Rupprechter G., Freund H.-J., Lennon D. (2005). The application of infrared spectroscopy to probe the surface morphology of alumina-supported palladium catalysts. J. Chem. Phys..

[58] Binet C., Jadi A., Lavalley J.-C. (1989). Évaluation de l’importance relative des faces cristallographiques du palladium supporté sur alumine: Étude infrarouge à l’aide de la molécule sonde CO. J. Chim. Phys..

[59] Davydov A., Sheppard N.T. (2003). Molecular Spectroscopy of Oxide Catalyst Surfaces.

[60] Angell C.L., Schaffer P.C. (1966). Infrared Spectroscopic Investigations of Zeolites and Adsorbed Molecules. II. Adsorbed Carbon Monoxide1. J. Phys. Chem..

[61] Ballivet-Tkatchenko D., Coudurier G. (1979). Adduct formation and further reactivity of iron carbonyl complexes introduced into a zeolite matrix. Inorg. Chem..

[62] Couble J., Bianchi D. (2011). Heats of adsorption of linear CO species adsorbed on reduced Fe/Al_2_O_3_ catalysts using the AEIR method in diffuse reflectance mode. Appl. Catal. A Gen..

[63] Fellah M.F. (2011). CO and NO Adsorptions on Different Iron Sites of Fe-ZSM-5 Clusters: A Density Functional Theory Study. J. Phys. Chem. C.

[64] Mihaylov M., Ivanova E., Chakarova K., Novachka P., Hadjiivanov K. (2011). Reduced iron sites in Fe–BEA and Fe–ZSM-5 zeolites: FTIR study of CO adsorption and 12C16O–13C18O co-adsorption. Appl. Catal. A Gen..

[65] Wielers A.F.H., Kock A.J.H.M., Hop C.E.C.A., Geus J.W., van Der Kraan A.M. (1989). The reduction behavior of silica-supported and alumina-supported iron catalysts: A Mössbauer and infrared spectroscopic study. J. Catal..

[66] Lingaiah N., Sai Prasad P.S., Kanta Rao P., Berry F.J., Smart L.E. (2002). Structure and activity of microwave irradiated silica supported Pd–Fe bimetallic catalysts in the hydrodechlorination of chlorobenzene. Catal. Commun..

[67] Lieltz G., Nimz M., Völter J., Läzär K., Guczi L. (1988). Double promotion of palladium/silica catalysts by iron and magnesium oxide in the synthesis of methanol from carbon monoxide and hydrogen. Appl. Catal..

[68] Karski S., Witońska I., Rogowski J., Gołuchowska J. (2005). Interaction between Pd and Ag on the surface of silica. J. Mol. Catal. A Chem..

[69] Witonska I., Krolak A. (2011). Catalytic hydrodechlorination of 2,4-dichlorophenol over Pd/SiO_2_ and Pd-Bi/SiO_2_ systems. Effect of the chemical nature of silica. Przem. Chem..

[70] Gómez-Quero S., Cárdenas-Lizana F., Keane M.A. (2008). Effect of Metal Dispersion on the Liquid-Phase Hydrodechlorination of 2,4-Dichlorophenol over Pd/Al_2_O_3_. Ind. Eng. Chem. Res..

[71] Xia C., Liu Y., Zhou S., Yang C., Liu S., Xu J., Yu J., Chen J., Liang X. (2009). The Pd-catalyzed hydrodechlorination of chlorophenols in aqueous solutions under mild conditions: A promising approach to practical use in wastewater. J. Hazard. Mater..

[72] Zinovyev S., Shelepchikov A., Tundo P. (2007). Design of new systems for transfer hydrogenolysis of polychlorinated aromatics with 2-propanol using a Raney nickel catalyst. Appl. Catal. B Environ..

[73] Ukisu Y., Kameoka S., Miyadera T. (2000). Catalytic dechlorination of aromatic chlorides with noble-metal catalysts under mild conditions: Approach to practical use. Appl. Catal. B Environ..

[74] Ukisu Y., Iimura S., Uchida R. (1996). Catalytic dechlorination of polychlorinated biphenyls with carbon-supported noble metal catalysts under mild conditions. Chemosphere.

[75] Ukisu Y., Miyadera T. (1997). Hydrogen-transfer hydrodehalogenation of aromatic halides with alcohols in the presence of noble metal catalysts. J. Mol. Catal. A Chem..

[76] Li C., Hoffman M.Z. (1999). One-Electron Redox Potentials of Phenols in Aqueous Solution. J. Phys. Chem. B.

[77] Pavitt A.S., Bylaska E.J., Tratnyek P.G. (2017). Oxidation potentials of phenols and anilines: Correlation analysis of electrochemical and theoretical values. Environ. Sci. Process. Impacts.

[78] Tanwongwan W., Eiad-ua A., Kraithong W., Viriya-empikul N., Suttisintong K., Klamchuen A., Kasamechonchung P., Khemthong P., Faungnawakij K., Kuboon S. (2019). Simultaneous activation of copper mixed metal oxide catalysts in alcohols for gamma-valerolactone production from methyl levulinate. Appl. Catal. A Gen..

[79] Kulkarni D., Wachs I.E. (2002). Isopropanol oxidation by pure metal oxide catalysts: Number of active surface sites and turnover frequencies. Appl. Catal. A Gen..

