# Supplementary material for: Hydrodechlorination of 4-Chlorophenol on Pd-Fe Catalysts on Mesoporous ZrO2SiO2 Support
Source: Molecules. 2020 Dec 30;26(1):141. doi: 10.3390/molecules26010141 (PMC7795707; doi:10.3390/molecules26010141)
Supplement: Supplementary file 1 [file molecules-26-00141-s001.pdf]

Supplementaly material

Hydrodechlorination of 4-Chlorophenol on Pd-Fe Catalysts on Mesoporous ZrO_2SiO_2 Support

Ekaterina S. Lokteva [1,*], Vera V. Shishova [1], Nikolay N. Tolkachev [2], Andrey N. Kharlanov [1], Konstantin I. Maslakov [1], Alexey O. Kamaev [1], Igor Yu. Kaplin [1], Irina N. Savina [3], and Elena V. Golubina [1]

[1] Lomonosov Moscow State University, Chemistry Department, Leninskie Gory 1/3, Moscow 119991, Russia; vshishova@bk.ru (V.V.S.); kharl@kge.msu.ru (A.N.K.); Nonvitas@gmail.com (K.I.M.); kamaevalexey@gmail.com (A.O.K.); kaplinigormsu@gmail.com (I.Y.K.); golubina@mail.ru (E.V.G.)

[2] N.D. Zelinsky Institute of Organic Chemistry, Russian Academy of Sciences, Leninsky Prospect 47, Moscow 119991, Russia; nick33@bk.ru

[3] School of Pharmacy and Biomolecular Sciences, University of Brighton, Huxley Building, Lewes Road BN2 4GJ, Brighton BN2 4GJ, UK; i.n.savina@brighton.ac.uk

* Correspondence: e.lokteva@rambler.ru; Tel.: +7-916-780-3363

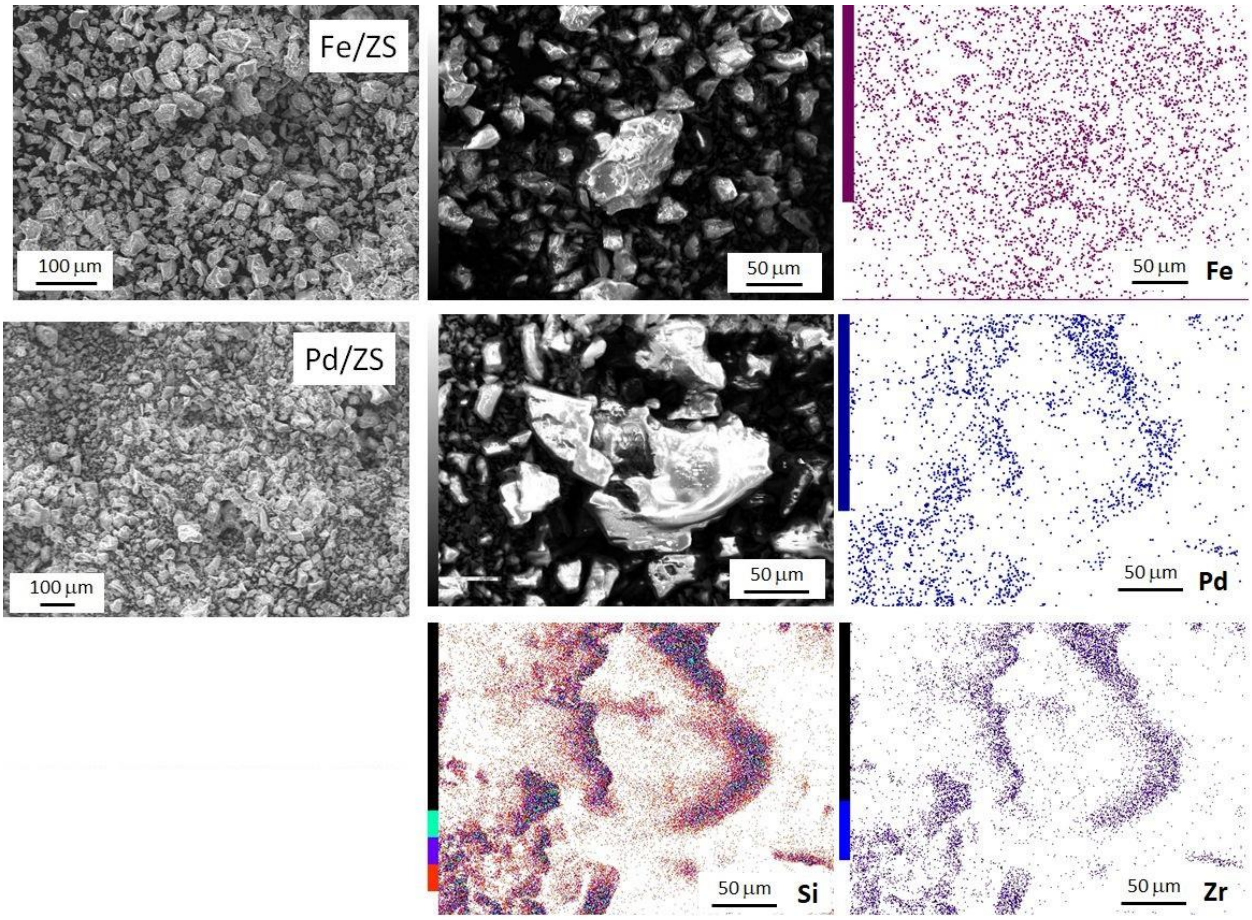

Figure S1: SEM images of Pd/ZS and Fe/ZS (after reduction with H_2 at 320 and 500 ^oC, respectively); the maps of elements distribution on the surface produced by EDS.

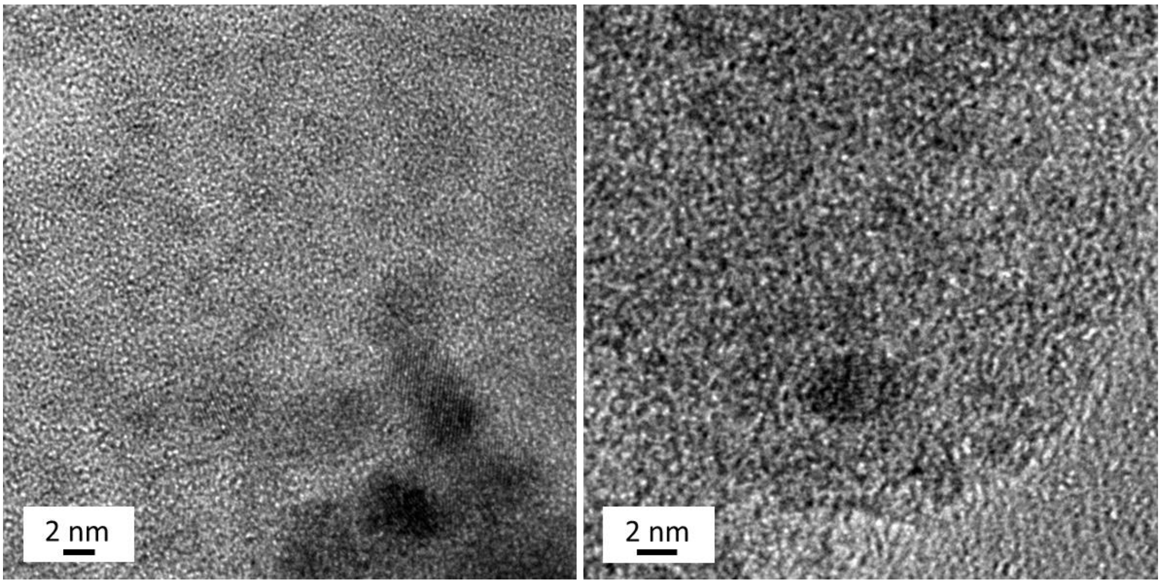

Figure S2. HR TEM images for Pd/ZS after the simultaneous treatment with water solution of phenol (75 mg/L) and H_2 (10 mL/min) at 30°C (1 h)

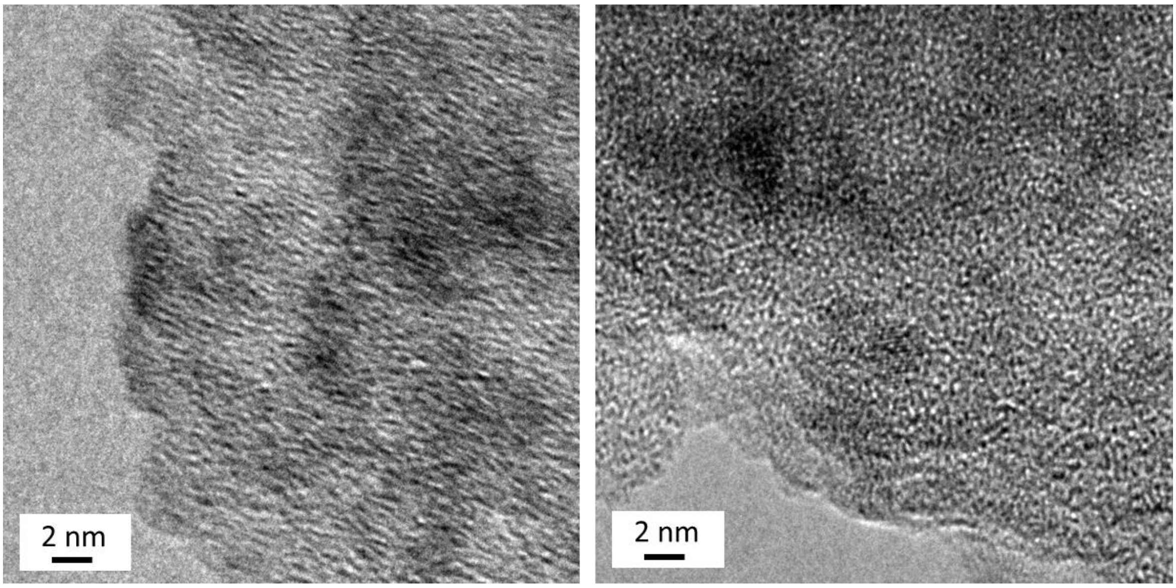

Figure S3. HR TEM images for FePd/ZS after the treatment with H_2 (10 mL/min) in water at 30°C (1 h)

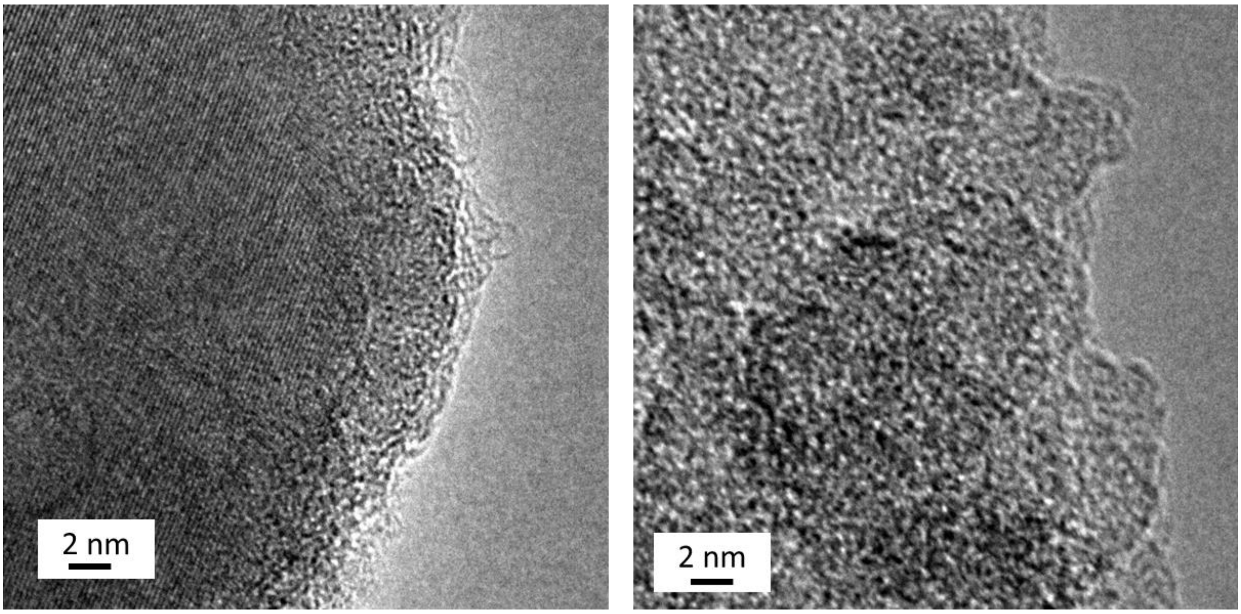

Figure S4. HR TEM images for Fe/ZS after the simultaneous treatment with water solution of phenol (75 mg/L) and H_2 (10 mL/min) at 30°C (1 h)

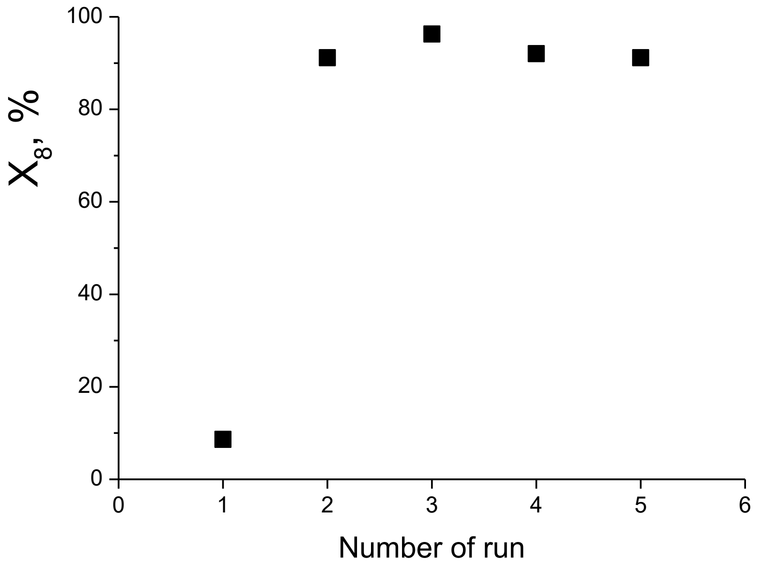

Figure S5. The conversion of 4-PhCl after 8 min of reaction on Pd/ZS in five consecutive runs. The preliminary reduction before the first run was performed using aqueous solution of $NaBH_4$ and H_2

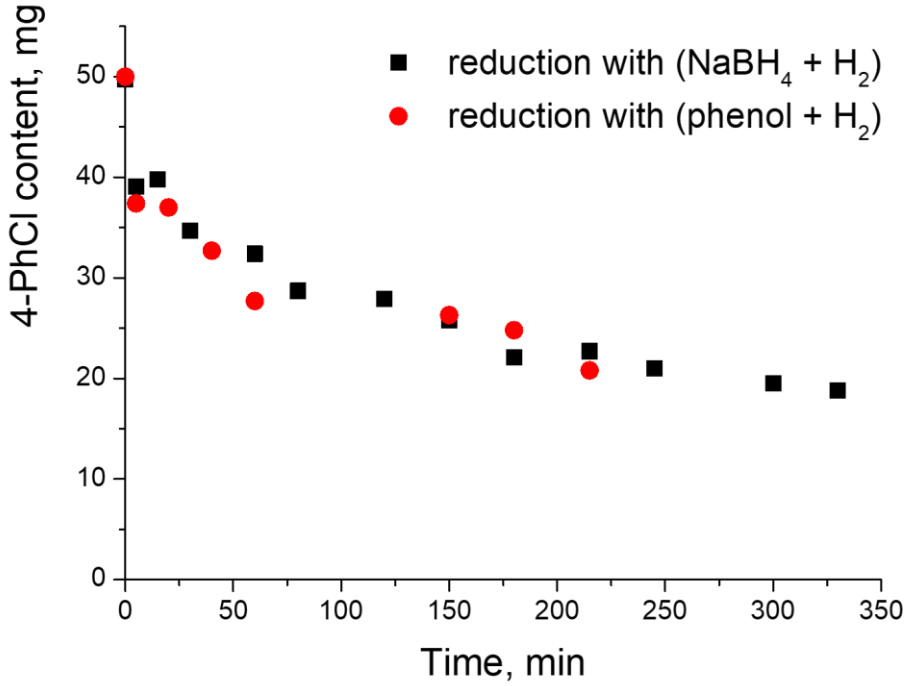

Figure S6. A comparison of Fe/ZS catalytic performance after reduction with aqueous solution of $NaBH_4$ and H_2 + phenol in aqueous solution, both treatments were performed at RT, 1 h
